# Household air pollution from solid fuel use as a dose-dependent risk factor for cognitive impairment in northern China

**DOI:** 10.1038/s41598-022-10074-6

**Published:** 2022-04-13

**Authors:** Tzu-Wei Joy Tseng, Ellison Carter, Li Yan, Queenie Chan, Paul Elliott, Majid Ezzati, Frank Kelly, James J. Schauer, Yangfeng Wu, Xudong Yang, Liancheng Zhao, Jill Baumgartner

**Affiliations:** 1 Department of Epidemiology, Biostatistics and Occupational Health, McGill University, 1130 Pine Ave W, Montreal, QC H3A 1A3 Canada; 2 Institute on the Environment, University of Minnesota, Saint Paul, MN USA; 3 Department of Civil and Environmental Engineering, Colorado State University, Fort Collins, CO USA; 4 Department of Epidemiology and Biostatistics, School of Public Health, Imperial College London, London, UK; 5 Environmental Research Group, MRC Centre for Environment and Health, School of Public Health, Imperial College London, London, UK; 6 MRC Centre for Environment and Health, School of Public Health, Imperial College London, London, UK; 7 NIHR Imperial College London Biomedical Research Centre, London, UK; 8 Department of Civil and Environmental Engineering, University of Wisconsin, Madison, USA; 9 Environmental Chemistry & Technology Program, University of Wisconsin, Madison, USA; 10 Peking University Clinical Research Institute, Beijing, China; 11 Department of Building Science, School of Architecture, Tsinghua University, Beijing, China; 12 National Center for Cardiovascular Disease, Fuwai Hospital, Peking Union Medical College & Chinese Academy of Medical Sciences, Beijing, China; 13 Institute for Health and Social Policy, McGill University, Montreal, Canada

**Keywords:** Energy and society, Dementia, Risk factors

## Abstract

The relationship between exposure to household air pollution (HAP) from solid fuel use and cognition remains poorly understood. Among 401 older adults in peri-urban northern China enrolled in the INTERMAP-China Prospective Study, we estimated the associations between exposure to HAP and z-standardized domain-specific and overall cognitive scores from the Montreal Cognitive Assessment. Interquartile range increases in exposures to fine particulate matter (53.2-µg/m^3^) and black carbon (0.9-µg/m^3^) were linearly associated with lower overall cognition [− 0.13 (95% confidence interval: − 0.22, − 0.04) and − 0.10 (− 0.19, − 0.01), respectively]. Using solid fuel indoors and greater intensity of its use were also associated with lower overall cognition (range of point estimates: − 0.13 to − 0.03), though confidence intervals included zero. Among individual cognitive domains, attention had the largest associations with most exposure measures. Our findings indicate that exposure to HAP may be a dose-dependent risk factor for cognitive impairment. As exposure to HAP remains pervasive in China and worldwide, reducing exposure through the promotion of less-polluting stoves and fuels may be a population-wide intervention strategy to lessen the burden of cognitive impairment.

## Introduction

Cognitive impairment, commonly symptomatic of dementia, is a leading cause of disability and dependency among older people, posing large socioeconomic and health burdens^1^. An estimated 50 million people worldwide have dementia, nearly a quarter of whom live in China^2^. Global healthcare expenditures for dementia are ~ US $1 trillion annually and increasing with disease burden^1^. Global prevalence of dementia is projected to triple to 152 million by 2050 as life expectancies increase in low- and middle-income countries^1^.

Epidemiological data reveal that while aging increases the risk of developing dementia, aging does not inevitably cause dementia and some risk is modifiable^3^. Identifying novel modifiable risk factors for cognitive impairment can inform more effective intervention strategies and health policies, and is a global health priority^1^. The most established risk factors for cognitive impairment include less education, chronic health conditions (e.g., cerebrovascular disease, diabetes), and behaviours such as tobacco use, alcohol consumption, physical inactivity, and social isolation^1^. More recently, attention has turned to environmental factors including air pollution. In addition to its well-established cardio-pulmonary impacts^4^, an increasing number of studies associate long-term exposure to outdoor air pollution with cognitive impairment and dementia^5^. In 2020, the *Lancet* Commission on Dementia added urban air pollution to its list of modifiable risk factors for dementia^1^.

Less understood is whether exposure to household air pollution (HAP) emitted from solid fuel (coal/biomass) stoves also reduces cognition. Almost half (49%) of the world’s population, including over 450 million Chinese, primarily use highly-polluting solid fuel stoves for cooking or space-heating^6^. Studies in China^7–12^, India^13^, Mexico^14^, and Ireland^15^ observed worse cognition among adults using solid fuel stoves or fireplaces. These studies motivate exposure–response investigations that estimate the cognitive impacts of air pollution from solid fuel stoves across a range of exposures.

We investigated the associations between HAP and cognition in Chinese adults enrolled in the International Study of Macro/Micronutrients and Blood Pressure (INTERMAP)-China Prospective (ICP) Study, a multi-provincial study that included measurement of personal exposures to fine particulate matter (PM_2.5_) and black carbon, household fuel use, cognition assessed by the Montreal Cognitive Assessment (MoCA; tested and validated in an elderly Chinese population that was similar to our study participants^16,17^), and a comprehensive set of covariates^18^.

## Results

Our analysis includes 401 participants from peri-urban Beijing and Shanxi without a history of stroke and who completed air pollution and cognitive assessments (Methods and Supplementary Fig. 1). Mean participant age was 62.5 years at the first visit and 58% were female (Table 1). Most were subsistence farmers (77%) and had at least primary school education (83%). A quarter (24%) of participants were tobacco smokers; among non-smokers, 21% were former smokers and 58% lived with one or more smokers. Over half (53%) had hypertension, 14% had diabetes, and 7% had heart disease (Supplementary Table 1). Overall MoCA scores ranged from 2 to 30 points (mean: 20.6) before adjustment for education (Table 2). Participants cooking with solid fuel stoves scored lower than clean fuel users in four of seven cognitive domains and for overall cognition.

**Table 1:** Characteristics of study participants by current cooking fuel use.

Characteristic	Exclusive use of clean fuel cookstoves (n = 220)	Use of solid fuel cookstoves (n = 180)	All participants (n = 401)^a^
Province; n (%)			
Beijing	137 (62)	64 (36)	202 (50)
Shanxi	83 (38)	116 (64)	199 (50)
Age (years); mean (SD)	61.7 (8.4)	63.5 (7.3)	62.5 (8.0)
Female; n (%)	129 (59)	102 (57)	232 (58)
Education level; n (%)			
No school	34 (15)	31 (17)	66 (16)
Primary school	74 (34)	88 (49)	162 (40)
Secondary school/college	112 (51)	61 (34)	173 (43)
Occupation; n (%)			
Agriculture	172 (78)	135 (75)	308 (77)
Other job outside of the household	15 (7)	12 (7)	27 (7)
Not working outside of the household	33 (15)	33 (18)	66 (16)
Annual household income (RMB); n (%)			
< 2,500	12 (5)	28 (16)	40 (10)
2,500–4,999	17 (8)	15 (8)	33 (8)
5,000–9,999	31 (14)	22 (12)	53 (13)
10,000–19,999	37 (17)	40 (22)	77 (19)
20,000–34,999	40 (18)	34 (19)	74 (18)
≥ 35,000	53 (24)	22 (12)	75 (19)
Missing	30 (14)	19 (11)	49 (12)
Marital status; n (%)			
Married	206 (94)	156 (87)	362 (90)
Single, widowed, or divorced	14 (6)	24 (13)	39 (10)
Number of household occupants; mean (SD)	3.0 (1.5)	3.4 (2.0)	3.2 (1.7)
Self-reported health status; n (%)			
Excellent	23 (10)	24 (13)	47 (12)
Good	68 (31)	66 (37)	135 (34)
Fair	101 (46)	64 (36)	165 (41)
Poor	28 (13)	26 (14)	54 (13)
Smoking status^b^; n (%)			
Current smoker	46 (21)	49 (27)	95 (24)
Former smoker	38 (17)	27 (15)	65 (16)
Never smoker	136 (62)	104 (58)	241 (60)
Ever lived with a smoker for six months^b^; n (%)			
Never	33 (15)	30 (17)	63 (16)
Yes, but not now	37 (17)	28 (16)	66 (16)
Yes, at present	66 (30)	46 (26)	112 (28)
Frequency of farming; n (%)			
None	116 (53)	72 (40)	189 (47)
Sometimes	54 (25)	75 (42)	129 (32)
Daily	50 (23)	33 (18)	83 (21)
Frequency of exercising; n (%)			
None	84 (38)	84 (47)	168 (42)
Sometimes	48 (22)	52 (29)	100 (25)
Daily	88 (40)	44 (24)	133 (33)
Frequency of drinking alcohol; n (%)			
Never or stopped drinking in past year	144 (65)	113 (63)	258 (64)
Sometimes	53 (24)	47 (26)	100 (25)
Daily	23 (10)	20 (11)	43 (11)
Total cholesterol; mean (SD)	4.8 (1.0)	4.7 (1.0)	4.7 (1.0)
Missing; n (%)	16 (7)	7 (4)	23 (6)
Had experienced past food shortage; n (%)	151 (69)	147 (82)	299 (75)

*SD* standard deviation, *RMB* Renminbi, *IQR* interquartile-range.

^a^Includes 1 participant with measured personal exposure to air pollution but missing fuel use data.

^b^Only never smokers reported whether they lived with a smoker. For statistical analysis, we constructed the following variables: current smoker, former smoker, never smoker who lived with a smoker, and no history of smoking or living with a smoker.

**Table 2:** Description of cognitive domains and associated tasks in the Montreal Cognitive Assessment (MoCA) survey and participant cognitive scores by current cooking fuel use.

Cognitive domain	Task description	Mean (SD)		
		Exclusive use of clean fuel cookstoves (n = 220)	Use of solid fuel cookstoves (n = 180)	All participants (n = 401)^a^
Visuospatial/executive	Matching five numbers (1–5) with corresponding Chinese numerals and tracing them in ascending order (1 point); Copying a three-dimensional cube (1 point); Drawing a clock that shows ten minutes after eleven (3 points)	0.1 (0.9)	0.0 (1.0)	0.1 (1.0)
Naming	Naming a lion, an elephant and a camel from the drawing (3 points)	0.0 (0.9)	0.1 (0.9)	0.1 (0.9)
Attention	Repeating a five-number sequence as heard and repeating in the backwards order a three-number sequence heard (2 points); Clapping hands only when hearing one from a sequence of random numbers (1 point); Subtracting seven from 100 and keep subtracting from the previous answer (3 points)	0.2 (0.9)	− 0.1 (1.0)	0.1 (0.9)
Language	Repeating two sentences exactly as heard (2 points); Telling as many different kinds of animals as possible in one minute (1 point)	0.0 (1.0)	0.1 (1.0)	0.0 (1.0)
Abstraction	Explaining what each pair of words have in common (e.g., orange and banana are both fruits) for two pairs of words (i.e., train and bicycle; watch and ruler) (2 points)	0.2 (1.0)	− 0.1 (1.0)	0.0 (1.0)
Delayed recall	Recalling five words that were asked to remember earlier freely without any cues (5 points)	0.1 (1.0)	0.0 (1.0)	0.1 (1.0)
Orientation	Telling the exact date and place of interview (6 points)	0.0 (0.9)	0.1 (0.8)	0.1 (0.9)
Overall				
z-score	Summing up scores from above seven domains for a possible maximum 30 points	0.1 (1.0)	0.0 (0.9)	0.1 (0.9)
Raw score^b^		20.9 (5.7)	20.3 (5.1)	20.6 (5.5)

All scores are standardized to z scores [mean (SD) = 0 (1)] to allow for comparability across domains.

*MoCA* montreal cognitive assessment.

^a^Includes 1 participant with measured personal exposure to air pollution but missing fuel use data.

^b^We did not add 1 point for participants with < 12 years of education as is standard for cognitive screening^16^ and instead adjusted for educational attainment in the statistical analysis.

More participants used solid fuels for heating (62%) compared with cooking (45%), and 22% used clean fuels exclusively for both (Supplementary Table 1). Compared with exclusive users of clean fuel, participants cooking with solid fuel were on average of older age, lower education and lower income, lived in households with more occupants, and more likely to be widowed (Table 1).

Estimated yearly personal exposures to PM_2.5_ and black carbon were moderately correlated (Spearman r = 0.46) and ranged from 17.2 to 484.4 µg/m^3^ (geometric mean, GM: 91.3; interquartile-range, IQR: 53.2) and 0.1–8.5 µg/m^3^ (GM: 1.4; IQR: 0.9), respectively (Supplementary Table 1 and Supplementary Fig. 2). Most participants (97%) were compliant in wearing the air samplers based on pedometer steps.

**Figure 1:**
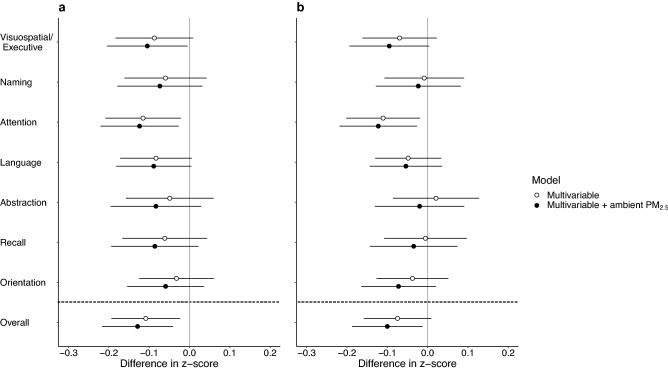
Associations between measures of cognitive function and personal exposures to air pollution in peri-urban northern Chinese adults. (**a,b**) Analyses were performed based on the data of 355 participants. Results from multivariable regression models, with final models (black circles) additionally adjusted for ambient PM_2.5_. Difference in z-score represents the difference in cognitive score associated with an IQR increase in exposure. Data are presented as point estimates of effects (central dots of the error bar) with 95% CIs (corresponding solid lines). The vertical solid line is the reference line. The horizontal dashed line separates estimate for overall cognition from estimates for cognitive domains. (**a**) Personal exposure to PM_2.5_ (IQR: 53.2 μg/m^3^) and MoCA scores. (**b**) Personal exposure to black carbon (IQR: 0.9 μg/m^3^) and MoCA scores. For detailed estimates of univariable and multivariable regression models, please see Supplementary Table 2.

In multivariable models, an IQR increase in PM_2.5_ exposure was associated with lower overall cognition [z-score: − 0.11 (95% confidence interval, CI: − 0.19, − 0.02)] and with lower domain-specific cognitive outcomes (Fig. 1a and Supplementary Table 2). Among the individual domains, attention had the largest inverse association with PM_2.5_ [− 0.12 (95% CI: − 0.21, − 0.02)]. Further adjusting for outdoor air quality resulted in slightly larger associations. We observed similar but slightly smaller coefficients for models with exposure to black carbon (Fig. 1b and Supplementary Table 2).

**Figure 2:**
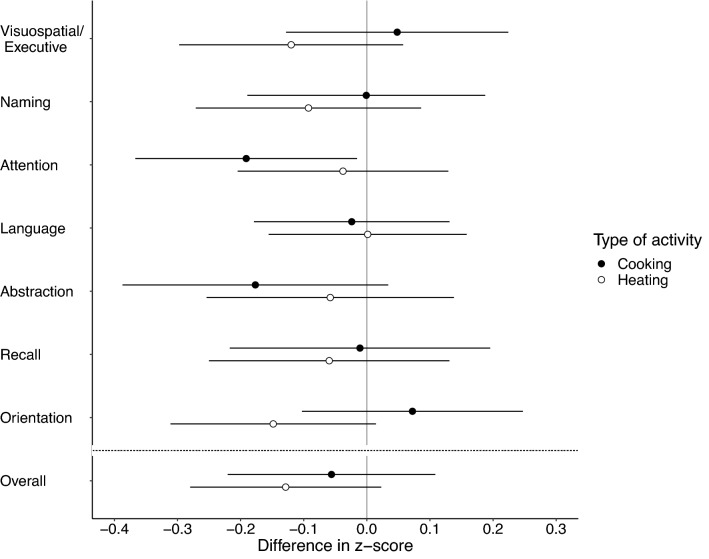
Associations between measures of cognitive function and current fuel use type in peri-urban northern Chinese adults. Analyses were performed based on the data of 400 participants. Results from multivariable regression models. Difference in z-score represents the difference in cognitive score associated with use of solid fuel for cooking (black circles) or heating (white circles) with exclusive use of clean fuel as reference. Data are presented as point estimates of effects (central dots of the error bar) with 95% CIs (corresponding solid lines). The vertical solid line is the reference line. The horizontal dashed line separates estimate for overall cognition from estimates for cognitive domains. For detailed estimates of univariable and multivariable regression models, please see Supplementary Table 2.

**Figure 3:**
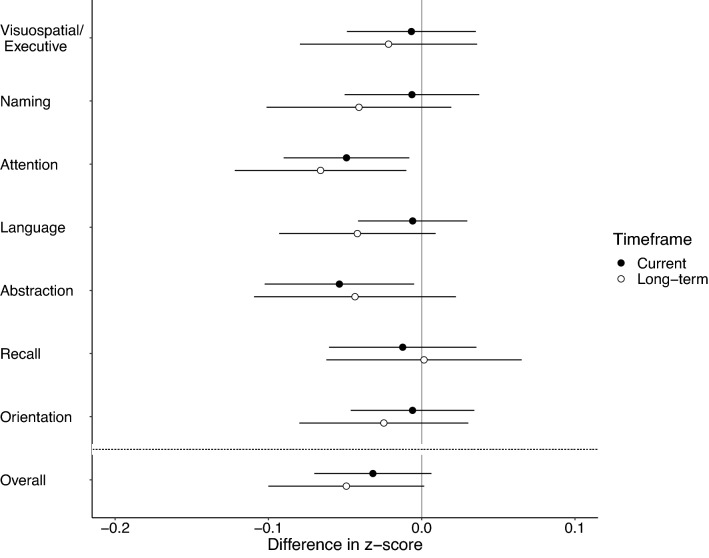
Associations between measures of cognitive function and intensity of indoor solid fuel use in peri-urban northern Chinese adults. Analyses were performed based on the data of 400 participants for intensity of indoor solid fuel use in the year prior to survey and 394 participants for intensity of indoor solid fuel use cumulatively over the past 20 years. Results from multivariable regression models. Difference in z-score represents the difference in cognitive score associated with a 100-day increase in solid fuel stove-use days in the past year (current; black circles) or a 5-year increase in solid fuel stove-use years over the past 20 years (long-term; white circles). Data are presented as point estimates of effects (central dots of the error bar) with 95% CIs (corresponding solid lines). The vertical solid line is the reference line. The horizontal dashed line separates estimate for overall cognition from estimates for cognitive domains. For detailed estimates of univariable and multivariable regression models, please see Supplementary Table 2.

Use of solid (versus exclusive clean) fuels for cooking or heating was associated with lower overall and most domain-specific cognitive outcomes in multivariable models (Fig. 2 and Supplementary Table 2), with attention for cooking (− 0.19, 95% CI: − 0.37, − 0.02) and orientation for heating (− 0.15, 95% CI: − 0.31, 0.01) having the largest associations. Current and long-term intensities of indoor solid fuel use were associated with lower overall and most domain-specific cognitive outcomes (Fig. 3 and Supplementary Table 2), particularly for attention [per 100-day increase in solid fuel stove-use days in the past year: − 0.05 (95% CI: − 0.09, − 0.01); per 5-year increase in solid fuel stove-use years over the past 20 years: − 0.07 (95% CI: − 0.12, − 0.01)].

**Figure 4:**
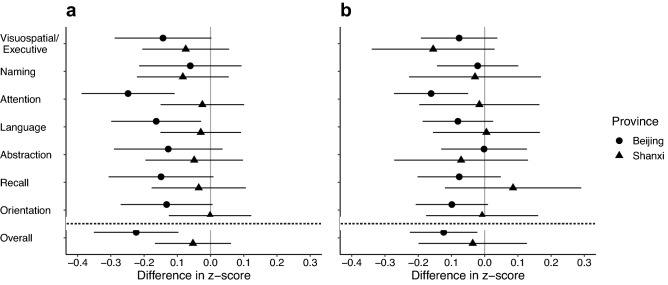
Associations between measures of cognitive function and personal exposures to air pollution in peri-urban northern Chinese adults, stratified by province of residence. (**a,b**) Analyses were performed based on the data of 355 participants. Results from multivariable regression models with province included as an interaction term with exposure variables. Difference in z-score represents the difference in cognitive score associated with an IQR increase in exposure. Data are presented as point estimates of effects (central dots of the error bar with circle representing Beijing and triangle representing Shanxi) with 95% CIs (corresponding solid lines). The vertical solid line is the reference line. The horizontal dashed line separates estimate for overall cognition from estimates for cognitive domains. (**a**) Personal exposure to PM_2.5_ (IQR: 53.2 μg/m^3^) and MoCA scores. (**b**) Personal exposure to black carbon (IQR: 0.9 μg/m^3^) and MoCA scores. For detailed estimates and interaction *p*-values, please see Supplementary Table 3.

The associations between exposure to PM_2.5_ and lower cognition were larger among Beijing than Shanxi participants (*P*_interaction_ < 0.05 for attention and overall cognition) (Fig. 4 and Supplementary Table 3), though no obvious effect modification by region was observed for stove use exposure metrics (Supplementary Table 3). We did not find strong or consistent evidence of effect modification by gender (Supplementary Table 3) or by education (Supplementary Table 4), though the magnitude of inverse associations were slightly larger among participants without formal education.

Our results did not appreciably change after (1) additionally adjusting for comorbid conditions (i.e., diabetes, heart disease, or hypertension) and body mass index; (2) adjusting for income using a more resolved variable instead; (3) additionally adjusting for heating fuel types in cooking fuel models and cooking fuel types in heating fuel models; (4) using a composite exposure variable that combined current cooking and heating fuels, or (5) and excluding participants who reported no history of solid fuel use, likely due to reporting error (Supplementary Tables 5, 6 and 7).

## Discussion

We observed consistent associations between higher exposure to HAP and worse cognition in our study of northern Chinese adults, with some evidence of regional differences. Air pollution from solid fuel stoves is one of the world’s most pervasive environmental exposures^6^. Our findings complement existing studies showing that exposure to outdoor air pollution increases risk of cognitive impairment and dementia^1,5^, and provide new evidence that exposure to HAP may also lead to worse cognition, which can be symptomatic of dementia.

In our study, higher personal exposures to air pollution PM_2.5_ and black carbon were linearly associated with lower cognition across wide exposure ranges (PM_2.5_: 17.2–484.4 µg/m^3^; black carbon: 0.1–8.5 µg/m^3^). Our findings extend the results of previous exposure–response studies of outdoor air pollution at much lower PM_2.5_ concentrations (< 0.1 to 61.8 µg/m^3^) that also observed dose-dependent associations with increased risk of cognitive impairment (i.e., ranging from 3 to 13% per 5-µg/m^3^ increase in PM_2.5_) among adults in mostly Europe and North America^5^. To our knowledge, ours is the first exposure–response study of air pollution and cognition with pollution levels ranging from moderate to high and in settings of household solid fuel use, thus contributing to our understanding of the cognitive impacts of air pollution in Latin America, Sub-Saharan Africa, and Asia where PM_2.5_ levels and sources overlap with our study setting^19^.

Among our participants, a 100-µg/m^3^ increase in exposure to PM_2.5_ was associated with scoring 1.41-points lower in overall cognition in multivariable models. Based on our regression results and contextualizing them using methods employed in previous studies^7,14^, we estimate that a theoretical shift in yearly PM_2.5_ exposure from the WHO’s interim annual target of 35 μg/m^3^ to our study mean (101.8 µg/m^3^) is the equivalent to the cross-sectional lower cognition associated with aging 5.5 years in our study (Supplementary Table 8). A modeling study estimated that interventions delaying both the onset and progression of Alzheimer’s dementia by even one year could reduce the number of prevalent cases worldwide by about 9.2 million over 40 years^20^. Since HAP impacts nearly half the world’s population^6^, most of whom live in countries where the burden of dementia is increasing most rapidly^1,2^, our findings highlight potential global health benefits of large-scale HAP intervention strategies that promote overall and cardiovascular health^4,21^ may also help preserve cognitive function into later life.

Our air pollution-cognition results are further supported by our findings that users of solid fuel stoves had lower cognition than exclusive clean fuel users, and that greater intensity of solid fuel use in the past year and past two decades were also associated with lower overall cognition. These results align with previous cross-sectional studies in China^7–11^, India^13^, Mexico^14^, and Ireland^15^ that also observed worse adult cognition among users of solid fuel or kerosene stoves, as well as longitudinal analyses in China showing faster cognitive decline among solid fuel users compared with those who transitioned to gas or electric stoves^7,11,12^. Together with these studies, our results suggest that policies and energy programs that encourage households to adopt clean fuel stoves and decrease their use of solid fuels may yield the largest cognitive benefits.

Among individual cognitive domains, attention had the largest and most consistent adverse associations with HAP exposure in our study. This is a notable finding because the attention domain in MoCA is particularly discriminatory of Alzheimer’s disease^16^. Previous studies of solid fuel use in China, Mexico, and Ireland similarly observed significant associations with attention by itself^14^ or when aggregated with orientation^7^ as well as visuospatial function^10^, whereas associations with recall^9,11,14,15^ and naming^15^ were smaller and less consistent. Earlier studies did not standardize their cognitive test scores to allow for comparability of estimates across domains^7–11,14,15^, thus barring their ability to assess the extent to which whether some aspects of cognitive function are more vulnerable to HAP than others.

The exact biological mechanisms through which air pollution exposure affects cognition are unclear, though a direct causal effect is plausible^22^. Incomplete combustion including solid fuel burning generates PM_2.5_^6,19^, which can reach the brain via the olfactory route or by crossing the blood–brain barrier^22^. PM_2.5_ can exert its toxicity on the brain through mechanisms that include reactive oxygen species production, microglia activation, DNA damage, and Amyloid-β peptide precipitation that result in neuroinflammation, cognitive dysfunction, and dementia-resembling brain pathologies^22,23^. Exposure to air pollution could also indirectly affect cognition by increasing risks for hypertension and cardiovascular diseases^4,21,22^, which are risk factors for cognitive impairment and dementia^1,24^. However, adjusting for history of hypertension and heart disease did not substantially change the results in our study, suggesting that this may not have been the dominant pathway that explains the association between HAP and cognition.

The larger associations between PM_2.5_ and cognition among Beijing participants could be attributable to differences in genetics or other underlying health conditions between participants in Shanxi and Beijing, though the health outcomes assessed in this study were similar across sites (Supplementary Table 9) and we did not observe regional differences in models with fuel-based exposures. Regional differences in the chemical composition of PM_2.5_ from different combustion sources is another possible explanation. Coal stoves were commonly used at both sites, but biomass stoves were more commonly used in Beijing^25^.

Field studies of emissions from different solid fuel stove types in China show large differences in the levels and chemical composition of particles emitted from stoves that burn coal and different forms of biomass, which may differentially impact their effects on health^26,27^. In our study, we were unable to distinguish between the cognitive effects of biomass (wood, agricultural residues) versus coal stove use because most households using biomass also used coal and because chemical composition analysis of PM_2.5_ was conducted for only a sub-sample of exposures (~ 11%)^25^. Whether any use or intensity of use of different fuel types or the chemical composition of PM_2.5_ emitted from different sources modifies an association between HAP and cognition is a topic to consider for future studies.

China’s burden of dementia exceeds the global average and, in the absence of intervention, is expected to increase as its population ages^1,2^. Population-level interventions that prevent or delay the onset of dementia are urgently needed. Our study indicates that provision of clean household energy may be one solution. Transition to gas and electricity requires substantial investment in infrastructure and technology, and often long-term fuel subsidies. Fortunately, China is well-positioned to implement large-scale household energy programs. Hundreds of millions of Chinese households adopted gas or electric stoves over the past two decades^28^, and large-scale clean energy programs like northern China’s clean heating program that bans coal and subsidizes electric heaters^29^ may also confer cognitive health benefits and help mitigate burden of dementia.

Notable strengths of our study include measurement of personal exposures to air pollution over multiple days and seasons and detailed fuel use information over the last 20 years, which overlaps with the preclinical period before diagnosis of dementia^1^. Importantly, our results were consistent across air pollution and fuel use exposure metrics, which have different sets of confounders. We used a clinically relevant cognitive assessment tool that was rigorously tested and validated to detect mild cognitive impairment in older adults^16^, including in Chinese populations^17^. Finally, important known confounders not included in most previous studies of household stove use and cognition were measured in our study using validated instruments and standard procedures, and included in the statistical analysis.

Our study has several limitations to consider for future studies. First, although many potential confounders were statistically controlled for in the analyses, we cannot eliminate the possibility of bias due to residual confounding in our observational study. Specifically, we were unable to measure mental health factors (e.g., depressive symptoms) that can influence cognitive assessment, though they are unlikely to substantially bias the estimates and could also be along the causal pathway^30^. Second, our single cognitive assessment precluded us from evaluating HAP-related differences in the trajectories of cognitive decline and reverse causality is also possible, though small differences in cognitive outcomes are unlikely to affect long-term stove use behaviors. Third, this study was conducted in a non-random sample of ICP Study participants and selection bias is thus a possibility, though no meaningful differences in sociodemographic and health characteristics were observed between the analytic sample and all participants who were otherwise eligible (Supplementary Table 10). Finally, some degree of exposure misclassification is expected in our self-reported stove-use variables which were designed to estimate longer-term exposure to HAP. We were able to externally verify a sub-sample of self-reported data based on household visits and village records of electricity and gas use^28^, but could not externally verify all historical stoves use reports. While most misclassification in stove use or intensity of use is likely non-differential and would usually result in bias toward null, it is possible that people with greater cognitive decline were less able to accurately report their stove use, which could lead to bias. Though, underreporting of solid fuel exposure among participants with greater cognitive decline would likely result in bias toward the null. Further, our complementary analysis with personal exposure to PM_2.5_, which is not subject to the same errors as self-report, showed similar trends to models with subjective assessment of fuel use. We also assumed that intensity of use for a given stove remained constant over 5-year periods, which likely contributed to exposure misclassification due to changes in use or intensity of use during that period, though this error is likely non-differential and less likely to lead to bias away from the null.

Our study provides a novel contribution to understanding the cognitive impacts of HAP, which remains a pervasive environmental exposure that impacts over a billion people globally. Our results reinforce the importance of reducing exposures to HAP for non-communicable disease prevention and can be informative to stakeholders who work in healthy aging and are interested in characterizing the brain health benefits of air pollution mitigation strategies that encourage the adoption of clean energy and the de-intensification of solid fuel stove use.

## Methods

### Study setting

Our study took place in 14 villages located in peri-urban Beijing (Pinggu County: N40°8′, E117°6′) and Shanxi (Yu County: N38°05′, E113°24′) provinces, representing lower-income areas with energy use practices that are characteristic of northern China. Household use of highly-polluting biomass- and coal-fuelled stoves for cooking and space-heating was common^18,28^. More information about the study setting is provided elsewhere^18^.

### Study design and participants

The ICP Study is a longitudinal study that was established to identify environmental and nutritional risk factors for chronic disease. In 2015–16 we enrolled 547 adults in Beijing and Shanxi (aged 40–79 at enrolment, 56% female) into the study. Details on the study design and the sampling and recruiting of participants are described elsewhere^18^. Briefly, most participants (n = 398, aged 60–79 in 2015–16) were previously enrolled in the INTERMAP Study in 1995–97, which randomly sampled households in the study villages and then randomly selected one adult from each household to participate^18^. The remaining 149 participants (aged 40–59 in 2015–16) were selected at random from village rosters. The ICP Study also enrolled 235 adults in southern China, but cognition was not assessed. The present analysis includes 401 participants without a history of stroke and who completed air pollution and cognitive assessments (inclusion flowchart shown in Supplementary Fig. 1). We obtained written informed consent from participants and ethical approvals from all investigator institutions (McGill University: #A08-M37-16B; Imperial College London: #15IC3095; Peking University: #00,001,052–15,017; Tsinghua University: #20,140,077; Fuwai Cardiovascular Hospital: #2015–650). The protocol involving humans was performed in accordance with institutional guidelines and regulations.

Trained staff implemented the study measurements in Beijing in December 2015 and September 2016 and in Shanxi in August 2015 and November 2015^18^. We conducted two campaigns in all villages to capture the heating and non-heating seasons, which can impact environmental conditions and behaviors including household stove use^28^. In both campaigns, we administered structured questionnaires and measured air pollution and relevant covariates. In the second campaign, we collected blood samples and assessed cognition.

### Personal exposures to air pollution

We measured participants’ 24-h personal exposure to PM_2.5_ on 2 consecutive days in each campaign (96-h total) using the gold standard gravimetric method. Details about PM_2.5_ measurement and analysis are summarized here and published elsewhere^31^.

Participants wore waistpacks with air samplers that collected PM_2.5_ on Teflon filters. The air samplers consisted of Harvard Personal Exposure Monitors (H-PEMs) (Mesa Labs, Inc., USA) fitted with 37-mm polytetrafluoroethylene (PTFE) filters (Zefluor™; Pall Life Sciences, USA) with 2.0-μm pore size and connected to small pumps (Apex Pro or TUFF™; Casella Waste Systems, Inc., USA) operated at 1.8 L/min^32^. Pump flow rates were measured at the start and end of each sampling period using a field-calibrated rotameter (mini-BUCK Calibrator M-5, Buck Inc., USA). For quality control and potential contamination assessment, about 7% of field blank filters were placed inside identical H-PEMs, subjected to the same field conditions, and analyzed using the same protocol as the sample filters. Participants were instructed to perform routine daily activities but could place the samplers on an elevated surface within 2 m while sitting, sleeping, and bathing. We added pedometers to a random subsample of waistpacks (70% of 1788 measurements) to assess compliance and deemed participants with < 500 steps in 24-h as potentially non-compliant based on an observed cut-off in the pedometer data.

Filters were analysed for their PM_2.5_ mass. Before and after air sampling, the PTFE filters were conditioned in a temperature- and humidity-controlled environment for at least 24 h and weighed in duplicate for mass on a high-precision microbalance (MX-5; Mettler-Toledo, USA) at the Wisconsin State Laboratory of Hygiene. If the first two weights differed by > 15 μg, the filter was reweighed until a stable weight was achieved. The average of the closest two weights was used for analysis. The balance’s zero and span were checked after every batch of ten filters. Pre-sampling filter weights were subtracted from the post-sampling weights. We performed blank correction by subtracting season- and site-specific blank values for PM_2.5_ from the net filter weights and replaced negative blank-corrected mass by randomly assigning a value between 0 and half the limit of detection. We divided PM_2.5_ mass (μg) by the total volume of air (m^3^) that passed through the filters during 24-h sampling periods to obtain PM_2.5_ concentrations (μg/m^3^).

The filters were also analyzed for black carbon using an aethalometer (SootScan™ OT21 Transmissometer; Magee Scientific, USA)^33^. Black carbon is a component of PM_2.5_ emitted during incomplete combustion that has been more strongly associated with some health outcomes than the mass of PM_2.5_^34,35^. We performed further calibrations^31^ to equate the optical black carbon measurements to elemental carbon, and performed blank correction by subtracting the season- and site-specific blank values for black carbon from final optical attenuation values. We replaced negative blank-corrected mass loadings by randomly assigning a value between 0 and half the limit of detection. To obtain black carbon concentrations (μg/m^3^), we multiplied the corrected black carbon mass loadings (μg/cm^2^) by the area of each filter (9.03 cm^2^), then divided that mass by the total volume of air (m^3^) that passed through the filters during sampling.

Last, we estimated annual mean personal exposures to PM_2.5_ and black carbon by calculating a weighted average of season-specific exposures based on northern China’s long-established heating (4 months) and non-heating (8 months) seasons.

### Current and long-term indoor stove use

An image-based household energy questionnaire^28^ was used to construct a set of categorical and continuous stove-use variables that characterized current and long-term stove use and intensity of use. Briefly, participants identified all stoves ever used by their household over the past 20 years and reported, for each stove, the type of fuel used, purpose of use, location of use, duration of use in 5-year intervals, and frequency of use. Exclusive use of clean fuels refers to households using only gas or electric appliances. We used this information to construct the following variables: Current indoor stove use pattern for (1) cooking and (2) heating at the time of survey, and intensity of indoor solid fuel use (3) currently (past year) and (4) over the long-term (over the past 20 years). The development of these variables is described below.

*Current cooking or heating fuel use (i.e., exclusive use of clean fuels versus use of solid fuel stoves).* Each participant was classified into one of the following categories for current stove-use practices at the time of the survey: (1) exclusive use of clean fuel stoves, (2) use of solid fuel stoves indoors, (3) only outdoor use of solid fuel, and (4) no stove (applicable to variable for heating only). Participants who indicated only outdoor (n = 34) or rare use of solid fuel stoves during holidays or when hosting many people (n = 17 and 1 for cooking and heating, respectively) were classified as exclusive clean fuel users. Participants without a heating stove (n = 13) were categorized as exclusive clean fuel users for heating.

*Current intensity of solid fuel use (i.e., stove-use days in the past year).* For each stove used by the participant, we first collapsed frequency of use from 10 categories listed in the questionnaire^28^ to five categories: rare, heating season only, non-heating season only, weekly, daily. Then, for each indoor solid fuel stove currently used by the participant, we estimated the average number of stove-use days in the past year as follows:

- Rare (i.e., seldom, holidays, or when hosting many people) → 13 stove-use days per year based on the assumption of half a stove-use day for each statutory day off for a public holiday in China;
- Heating season (i.e., only in colder months) → 121 stove-use days per year based on the number of days in the heating season in northern China (November 15-March 15)^36^;
- Non-heating season (i.e., only in warmer months) → 244 stove-use days per year based on the number of days in the non-heating season in northern China (March 16-November 14);
- Weekly (i.e., three to four times per week; several days per week) → 182 stove-use days per year based on multiplying 3.5 days per week (i.e., the mid-point of 3 to 4 times per week) by 52 weeks per year
- Daily (i.e., 2–5, 14–16 or 24 h per day or everyday) → 365 stove-use days per year.

We used the same categories to estimate stove use intensity in Beijing and Shanxi, which are neighboring provinces with very similar public holidays, climates, and space heating needs^36^.

We next calculated current indoor solid fuel stove-use days for each participant as follows:

$$\mathop \sum \limits_{i = 1}^{n} ({\text{solid fuel stoves used indoors}}_{{\text{i}}} { } \times {\text{number of days used in the past year}})$$

where *i* is each solid fuel stove used indoors and *n* is the total number of solid fuel stoves used indoors. Participants exclusively using clean fuel stoves, using solid fuel stoves outdoors only, or with no stove (applicable to heating stoves only) were assigned a value of 0 solid fuel stove-use days.

*Long-term intensity of solid fuel use (i.e., stove-use years during the past 20 years).* For each stove used by participants, we collected information on when they started and stopped using it in 5-year intervals. For each indoor solid fuel stove used by the participant, we calculated the years of use since inception of the INTERMAP Study (20 years ago) as follow: midpoint year of time (in 5-year intervals) from when participants reported starting use of a stove to either the midpoint year of time (in 5-year intervals) that participant reported suspension of that stove or 0 if they reported still using it. For example, a participant who started using a stove 15 years ago and suspended use of the stove 5 years ago was assigned a duration of 10 years of use for that stove. Participants who reported starting and suspending use of a stove in the same 5-yr period were assigned a duration of 2.5 years of use for that stove (i.e., the midpoint of the 5-year interval).

Some participants did not report having a solid fuel cookstove during the past 20 years (n = 61), which we attribute to misreporting or data collection error since the original INTERMAP survey conducted in 1996 indicated that all households in the study villages cooked with solid fuel^28,37^. Informed by field observations and by cross-referencing survey responses with village records^28^, we assumed that these participants either used solid fuel stoves up until the time period that they reported regularly using clean fuel for cooking (n = 58) or were still using solid fuel stoves if no use of clean fuel stoves was reported (n = 3).

Finally, we combined information on duration of use and intensity of use (based on number of stove-use days per year categories described), and calculated total indoor solid fuel stove-use years during the past 20 years for each participant as follows:

$$\frac{{\begin{aligned} &{\mathop \sum \nolimits_{i = 1}^{n} ({\text{solid fuel stove used indoors}}_{{\text{i}}}} \\ & \quad \times {{\text{number of stove - use days per year for that stove}}} \\ & \quad \times {{\text{number of years used}})} \\ \end{aligned} }}{{365 \,{\text{days per year}}}}$$

where *i* is each solid fuel stove used indoors and *n* is the total number of solid fuel stoves used indoors.

### Assessment of cognitive function

Trained staff assessed cognition using the MoCA (https://www.mocatest.org/), a screening tool developed to detect mild cognitive impairment in middle-aged and older adults with high sensitivity and specificity^16,17^. MoCA evaluates seven individual cognitive domains: visuospatial/executive, naming, attention, language, abstraction, delayed recall, and orientation that yield domain-specific and overall scores (see Table 2 for task description and point system). Our pilot study identified four questions in the MoCA-Beijing survey that were linguistically or culturally inappropriate for our participants, reflecting issues previously observed^17^. We thus modified the questions using text from the Singapore and Changsha (China) versions of MoCA (changes shown in Supplementary Fig. 3).

### Covariates

The ICP Study administered household and individual questionnaires to collect information on household demographics, socioeconomic status, and chronic disease risk factors including alcohol consumption, tobacco use, secondhand smoking, physical activity, medical history, and past food shortage experiences^18^. Serum concentrations of triglycerides and high- and low-density lipoprotein cholesterol were analyzed using standard methods^18^.

Outdoor air pollution was assessed by inverse distance weighting the hourly PM_2.5_ data from government air monitoring stations (http://beijingair.sinaapp.com) within 50 km of each village and calculating 24-h averages that corresponded with personal exposure measurements. These estimates were highly correlated with village-level outdoor gravimetric PM_2.5_ measurements collected by the ICP Study on 24 study-days (Pearson r = 0.91)^31^.

### Statistical analysis

We summarized participants’ sociodemographic and health characteristics by current cooking fuel use. Mixed effects regression models with restricted maximum likelihood were used to estimate the cognitive associations with exposures to HAP. We specified a random effect at the village level and assumed a compound symmetry correlation structure given the relatively large number of participants clustered within villages and that the number of participants per village varied considerably (range: 1 to 74, median: 26)^38^. The general regression equation for the models is provided in the supplementary information (Supplementary Eq. 1).

For continuous exposures, we assessed the response function for overall raw cognitive score using natural cubic spline models with two to four degrees of freedom. All of these functions were deemed consistent with linearity through visual inspection (Supplementary Fig. 4). For cognitive outcomes, we z-standardized the domain-specific and overall raw MoCA scores (mean = 0; standard deviation = 1) to facilitate comparison of results across domains, but also maintained the raw score for overall cognition to allow for interpretation against the original survey.

Using directed acyclic graphs, we a priori identified known or suspected risk factors for cognitive impairment that were also plausibly associated with HAP without being on the causal pathway. The multivariable models were adjusted for age, gender, educational attainment, occupation, annual household income, exposure to tobacco smoking, frequency of exercising, frequency of farming, self-reported health status, frequency of drinking alcohol, marital status and number of household occupants as proxy measures of social contact^1,39^, total cholesterol and past experience with food shortage as proxy measures of diet and nutrition (cooking fuel models only)^1,24^, and province of residence (variable categories shown in Table 1). We additionally adjusted for outdoor PM_2.5_ in a second set of models with measured personal exposures to better isolate the exposure contribution of household stove use. Missing data for income (dichotomized as < or ≥ Renminbi 20,000; n = 49) and cholesterol (continuous; n = 23) were handled with multiple imputation as described elsewhere^28^.

We investigated potential effect modification by province, gender, and education based on findings in previous studies^8,9,11,12,15^. We also conducted multiple sensitivity analyses, including (1) adjusting for potential confounders that could also be along the causal pathway including co-morbid conditions (i.e., physician-diagnosed diabetes, heart disease, or hypertension) and body mass index^1,24^, (2) replacing the binary annual household income variable with a more resolved six-category income variable to examine potential residual confounding by income; (3) adjusting for heating fuel (for cooking fuel models) and cooking fuel (for heating fuel models); (4) combining current cooking and heating fuel use into a single exposure variable (i.e., any use of solid fuel stoves); (5) excluding the 61 participants who reported no history of solid fuel use during the past 20 years, which we believe was reporting error, from the analysis with ‘long-term intensity of use’ as the exposure.

All analyses were conducted in R version 4.0.3, using the “nlme”, “splines”, and “MICE” packages for mixed models, natural cubic splines, and multiple imputations, respectively.

## Supplementary Information

Supplementary Information.

## Data Availability

Requests for data that support the findings of this study will be reviewed and made available on a case-by-case basis by the study investigators, subject to compliance with Research Ethics Board restrictions for the survey data. Figures 1, 2, 3 and 4 and Supplementary Figs. 1, 2 and 4 contain primary data.

## Code availability

Requests for code generated and annotated in R to process and analyse the primary data collected in this study will be reviewed and made available upon reasonable request.
