# Supplementary material for: Household air pollution from solid fuel use as a dose-dependent risk factor for cognitive impairment in northern China
Source: Sci Rep. 2022 Apr 13;12:6187. doi: 10.1038/s41598-022-10074-6 (PMC9008006; doi:10.1038/s41598-022-10074-6)
Supplement: Supplementary file 1 — Supplementary Information. [file 41598_2022_10074_MOESM1_ESM.pdf]

Household air pollution from solid fuel use as a dose-dependent risk factor for cognitive impairment in northern China

Supplementary information

Tzu-Wei Joy Tseng,[1] Ellison Carter,[2,3] Li Yan,[4,5] Queenie Chan,[4,5,6] Paul Elliott,[4,6,7] Majid Ezzati,[4,6] Frank Kelly,[5] James J. Schauer,[8,9] Yangfeng Wu,[10] Xudong Yang,[11] Liancheng Zhao,[12] Jill Baumgartner,*[1,13]

[1]Department of Epidemiology, Biostatistics and Occupational Health, McGill University, Montreal, Quebec, Canada
[2]Institute on the Environment, University of Minnesota, Saint Paul, MN, USA
[3]Department of Civil and Environmental Engineering, Colorado State University, Fort Collins, CO, USA
[4]Department of Epidemiology and Biostatistics, School of Public Health, Imperial College London, London, UK
[5]Environmental Research Group, MRC Centre for Environment and Health, School of Public Health, Imperial College London, London, UK
[6]MRC Centre for Environment and Health, School of Public Health, Imperial College London, London, UK
[7]NIHR Imperial College London Biomedical Research Centre, London, UK
[8]Department of Civil and Environmental Engineering, University of Wisconsin, Madison, USA
[9]Environmental Chemistry & Technology Program, University of Wisconsin, Madison, USA
[10]Peking University Clinical Research Institute, Beijing, China
[11]Department of Building Science, School of Architecture, Tsinghua University, Beijing, China
[12]National Center for Cardiovascular Disease, Fuwai Hospital, Peking Union Medical College & Chinese Academy of Medical Sciences, Beijing, China
[13]Institute for Health and Social Policy, McGill University, Montreal, Canada

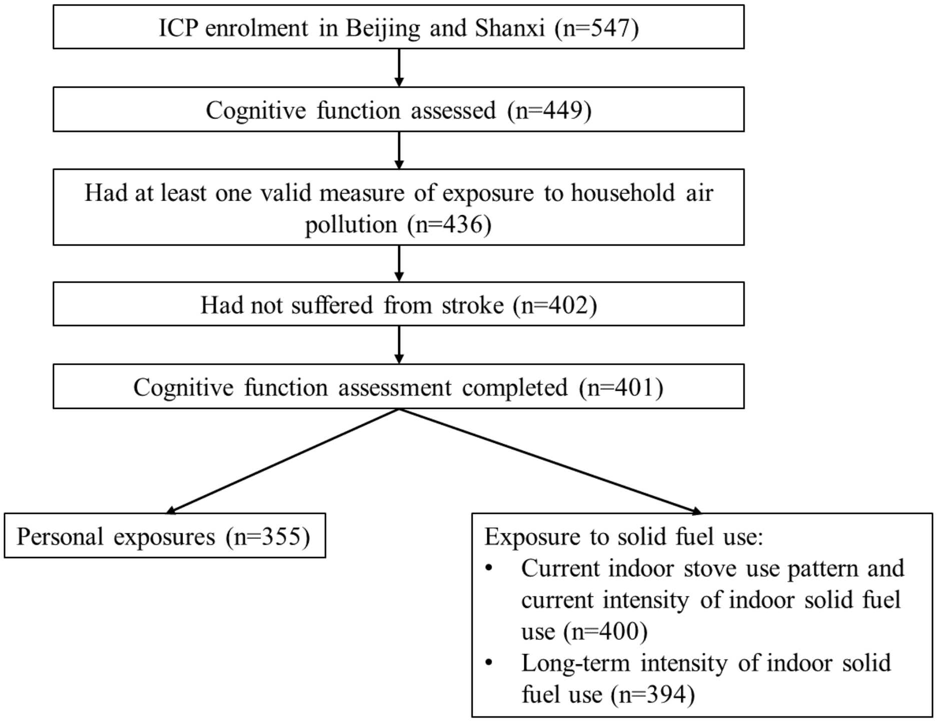

Supplementary Fig. 1. Inclusion flowchart of study participants. Refer to **Supplementary Table 10** for a comparison on baseline socio-demographic and health characteristics for ICP participants who would have been eligible for this study and the analytic sample.

Supplementary Table 1. Measures of exposure to household air pollution and health conditions among study participants by current cooking fuel use.

Characteristic	Exclusive use of clean fuel cookstoves (n=220)	Use of solid fuel cookstoves (n=180)	All participants (n=401)[a]
Personal exposure to $PM_{2.5}$ (μg/m^3)			
Mean (SD)	97.3 (51.6)	106.9 (51.2)	101.8 (51.6)
Median (IQR)	87.4 (52.1)	97.0 (60.0)	93.4 (53.2)
Geometric mean (95% CI)	86.9 (81.2, 93.0)	96.5 (90.0, 103.6)	91.3 (86.9, 95.9)
Missing; n (%)	28 (13)	18 (10)	46 (12)
Personal exposure to black carbon (μg/m^3)			
Mean (SD)	1.6 (0.9)	1.6 (0.8)	1.6 (0.9)
Median (IQR)	1.4 (0.9)	1.4 (0.9)	1.4 (0.9)
Geometric mean (95% CI)	1.4 (1.3, 1.5)	1.4 (1.3, 1.5)	1.4 (1.3, 1.5)
Missing; n (%)	28 (13)	18 (10)	46 (12)
Current heat stove use; n (%)			
Exclusive use of clean fuel	88 (40)	65 (36)	153 (38)
Use of solid fuel stoves	132 (60)	115 (64)	247 (62)
Missing	0 (0)	0 (0)	1 (0)
Current stove use type for both cooking and heating; n (%)			
Exclusive use of clean fuel	88 (40)	0 (0)	88 (22)
Use of solid fuel stoves	132 (60)	180 (100)	312 (78)
Missing	0 (0)	0 (0)	1 (0)
Current intensity of indoor solid fuel use (ie, stove-use days in the past year)			
Mean (SD)	83.0 (78.8)	433.3 (159.8)	240.6 (212.8)
Median (IQR)	121.0 (121.0)	486.0 (121.0)	121.0 (365.0)
Missing; n (%)	0 (0)	0 (0)	1 (0)
Long-term intensity of indoor solid fuel use (ie, stove-use years in the past 20 years)			
Mean (SD)	13.9 (6.6)	20.8 (7.4)	17.0 (7.8)
Median (IQR)	14.1 (10.0)	21.6 (5.8)	17.5 (12.5)
Missing; n (%)	5 (2)	1 (1)	7 (2)
Self-reported physician-diagnosed health conditions; n (%)			
Diabetes	33 (15)	22 (12)	55 (14)
Heart disease	16 (7)	13 (7)	30 (7)
Hypertension	120 (55)	94 (52)	214 (53)
Body mass index (kg/m^2); mean (SD)	26.4 (3.4)	25.8 (3.8)	26.1 (3.6)

Missing; n (%)	12 (6)	4 (2)	16 (4)

[a] Includes 1 participant with measured personal exposure to air pollution but missing fuel use data.

Abbreviations: $PM_{2.5}$, particulate matter ≤2.5 μm in aerodynamic diameter; SD, standard deviation; IQR, interquartile range; CI, confidence interval.

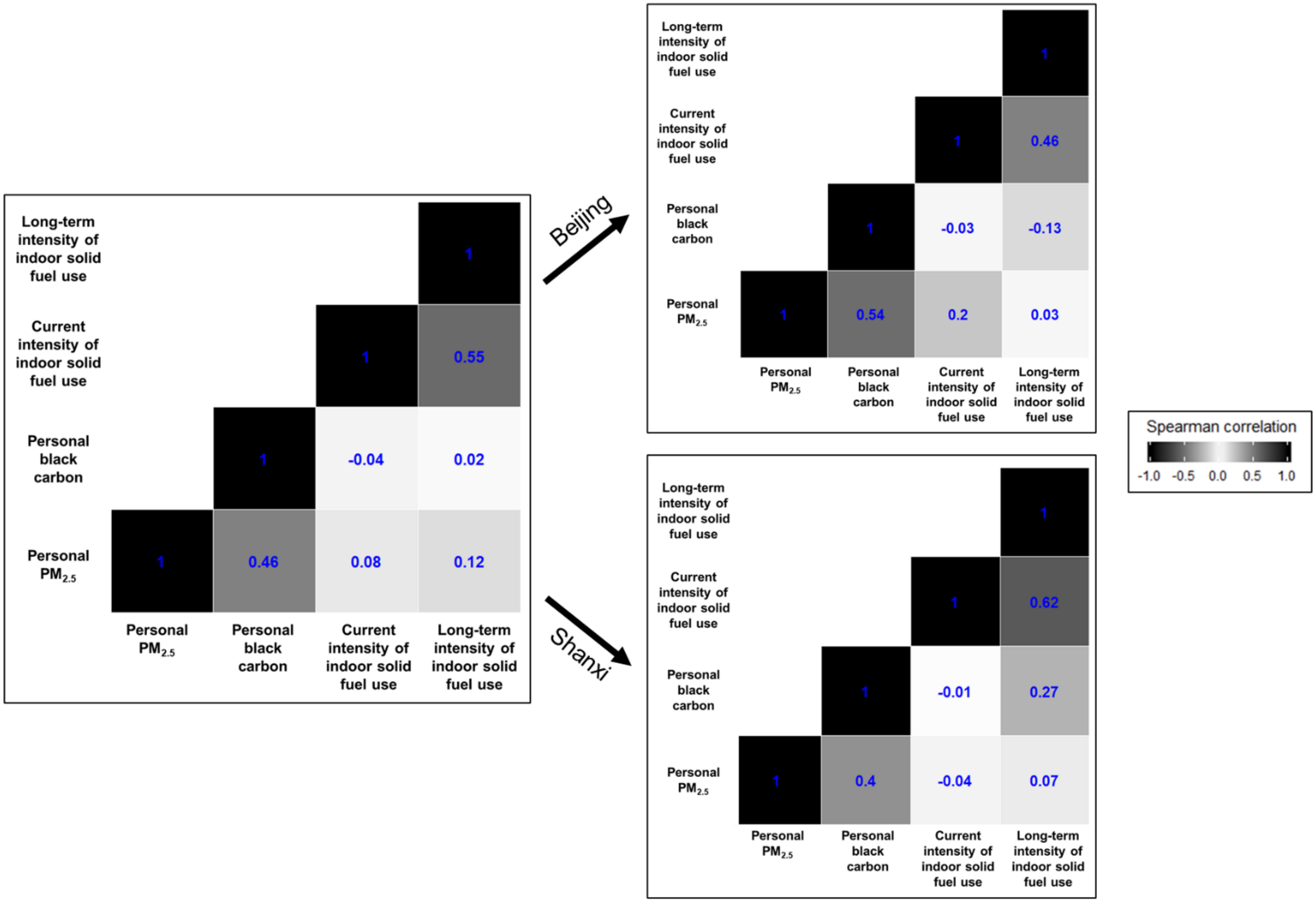

Supplementary Fig. 2. Spearman correlations between continuous exposure variables: personal exposures to $PM_{2.5}$ and black carbon ($\mu g/m^3$), current intensity of indoor solid fuel use (stove-use days in the past year), and long-term intensity of indoor solid fuel use (stove-use years in the past 20 years) for the entire study sample (n=401) and by province (n=202 in Beijing and 199 in Shanxi). Abbreviation: $PM_{2.5}$, particulate matter ≤2.5 μm in aerodynamic diameter.

Supplementary Table 2. Associations and 95% confidence intervals between measures of cognitive function and personal exposures to air pollution, current indoor fuel use patterns, or intensity of indoor solid fuel use: results from univariate and multivariable models.

		Univariable		Univariable plus age and gender		Univariable plus age, gender, and education		Multivariable		Multivariable plus ambient $PM_{2.5}$	
**Exposure**	**Cognitive domain**	**Difference in score[a]**	**95% CI**	**Difference in score[a]**	**95% CI**	**Difference in score[a]**	**95% CI**	**Difference in score[a]**	**95% CI**	**Difference in score[a]**	**95% CI**
Personal $PM_{2.5}$	Visuospatial/ Executive	0.01	(-0.18, 0.21)	-0.10	(-0.28, 0.08)	-0.12	(-0.29, 0.05)	-0.16	(-0.34, 0.02)	-0.20	(-0.38, -0.01)
	Naming	0.03	(-0.15, 0.22)	-0.05	(-0.23, 0.13)	-0.07	(-0.25, 0.12)	-0.11	(-0.30, 0.08)	-0.14	(-0.34, 0.06)
	Attention	-0.06	(-0.25, 0.12)	-0.15	(-0.33, 0.03)	-0.19	(-0.36, -0.03)	-0.22	(-0.39, -0.04)	-0.23	(-0.41, -0.05)
	Language	-0.12	(-0.28, 0.05)	-0.15	(-0.31, 0.02)	-0.18	(-0.34, -0.02)	-0.16	(-0.32, 0.01)	-0.17	(-0.34, 0.01)
	Abstraction	-0.02	(-0.22, 0.18)	-0.09	(-0.29, 0.10)	-0.12	(-0.31, 0.07)	-0.09	(-0.30, 0.11)	-0.16	(-0.37, 0.05)
	Delayed recall	-0.03	(-0.23, 0.17)	0.00	(-0.20, 0.19)	-0.04	(-0.23, 0.15)	-0.12	(-0.31, 0.08)	-0.16	(-0.37, 0.04)
	Orientation	-0.01	(-0.18, 0.16)	-0.01	(-0.19, 0.16)	-0.04	(-0.21, 0.12)	-0.06	(-0.23, 0.11)	-0.11	(-0.29, 0.07)
	Overall	-0.03	(-0.21, 0.15)	-0.10	(-0.27, 0.07)	-0.15	(-0.31, 0.00)	-0.20	(-0.36, -0.04)	-0.24	(-0.41, -0.08)
	Overall (raw score)	-0.18	(-1.24, 0.88)	-0.58	(-1.57, 0.41)	-0.89	(-1.79, 0.00)	-1.19	(-2.12, -0.25)	-1.41	(-2.37, -0.45)
Personal black carbon	Visuospatial/ Executive	-0.08	(-0.20, 0.04)	-0.07	(-0.18, 0.03)	-0.08	(-0.18, 0.03)	-0.08	(-0.18, 0.02)	-0.10	(-0.21, 0.00)
	Naming	0.00	(-0.11, 0.12)	-0.01	(-0.12, 0.09)	-0.01	(-0.12, 0.09)	-0.01	(-0.12, 0.10)	-0.03	(-0.14, 0.09)
	Attention	-0.13	(-0.24, -0.02)	-0.14	(-0.24, -0.03)	-0.13	(-0.23, -0.04)	-0.12	(-0.22, -0.02)	-0.13	(-0.24, -0.03)
	Language	-0.09	(-0.19, 0.01)	-0.09	(-0.20, 0.01)	-0.09	(-0.19, 0.00)	-0.05	(-0.14, 0.04)	-0.06	(-0.16, 0.04)
	Abstraction	0.01	(-0.12, 0.13)	-0.01	(-0.12, 0.11)	-0.01	(-0.12, 0.10)	0.02	(-0.09, 0.14)	-0.02	(-0.14, 0.10)
	Delayed recall	-0.06	(-0.18, 0.07)	-0.04	(-0.16, 0.07)	-0.05	(-0.16, 0.07)	-0.01	(-0.12, 0.11)	-0.04	(-0.16, 0.08)
	Orientation	-0.08	(-0.18, 0.03)	-0.07	(-0.18, 0.03)	-0.07	(-0.17, 0.03)	-0.04	(-0.14, 0.06)	-0.08	(-0.18, 0.02)
	Overall	-0.10	(-0.21, 0.01)	-0.11	(-0.21, -0.01)	-0.11	(-0.20, -0.02)	-0.08	(-0.17, 0.01)	-0.11	(-0.21, -0.01)
	Overall (raw score)	-0.60	(-1.25, 0.04)	-0.65	(-1.24, -0.06)	-0.66	(-1.19, -0.13)	-0.48	(-1.02, 0.06)	-0.64	(-1.20, -0.08)
Exclusive use of clean fuel for cooking (ref: use of solid fuel)	Visuospatial/ Executive	0.07	(-0.13, 0.28)	0.04	(-0.14, 0.22)	-0.03	(-0.20, 0.14)	-0.05	(-0.22, 0.13)	—	—
	Naming	-0.03	(-0.23, 0.17)	-0.04	(-0.23, 0.15)	-0.05	(-0.24, 0.14)	0.00	(-0.19, 0.19)	—	—
	Attention	0.21	(0.01, 0.41)	0.20	(0.02, 0.38)	0.16	(0.00, 0.33)	0.19	(0.02, 0.37)	—	—
	Language	0.07	(-0.12, 0.26)	0.04	(-0.14, 0.23)	0.03	(-0.16, 0.21)	0.02	(-0.13, 0.18)	—	—
	Abstraction	0.18	(-0.04, 0.40)	0.18	(-0.03, 0.39)	0.17	(-0.03, 0.37)	0.18	(-0.03, 0.39)	—	—
	Delayed recall	0.05	(-0.17, 0.27)	0.00	(-0.20, 0.21)	-0.04	(-0.24, 0.16)	0.01	(-0.20, 0.22)	—	—
	Orientation	-0.09	(-0.28, 0.10)	-0.11	(-0.30, 0.08)	-0.13	(-0.31, 0.05)	-0.07	(-0.25, 0.10)	—	—
	Overall	0.07	(-0.13, 0.27)	0.04	(-0.15, 0.22)	-0.01	(-0.17, 0.16)	0.06	(-0.11, 0.22)	—	—
	Overall (raw score)	0.40	(-0.76, 1.57)	0.21	(-0.85, 1.28)	-0.04	(-0.99, 0.91)	0.33	(-0.63, 1.29)	—	—

Exclusive use of clean fuel for heating (ref: use of solid fuel)	Visuospatial/ Executive	0.19	(-0.01, 0.39)	0.14	(-0.04, 0.33)	0.09	(-0.08, 0.27)	0.12	(-0.06, 0.30)	–	–
	Naming	0.14	(-0.05, 0.32)	0.13	(-0.04, 0.31)	0.12	(-0.06, 0.30)	0.09	(-0.09, 0.27)	–	–
	Attention	0.13	(-0.06, 0.32)	0.12	(-0.06, 0.29)	0.07	(-0.10, 0.23)	0.04	(-0.13, 0.20)	–	–
	Language	0.06	(-0.11, 0.24)	0.05	(-0.12, 0.22)	0.02	(-0.14, 0.18)	0.00	(-0.16, 0.16)	–	–
	Abstraction	0.07	(-0.14, 0.27)	0.05	(-0.14, 0.25)	0.02	(-0.17, 0.22)	0.06	(-0.14, 0.25)	–	–
	Delayed recall	0.10	(-0.10, 0.31)	0.10	(-0.10, 0.29)	0.05	(-0.14, 0.24)	0.06	(-0.13, 0.25)	–	–
	Orientation	0.18	(0.01, 0.35)	0.17	(0.01, 0.34)	0.15	(-0.01, 0.31)	0.15	(-0.01, 0.31)	–	–
	Overall	0.19	(0.01, 0.38)	0.18	(0.01, 0.35)	0.12	(-0.03, 0.27)	0.13	(-0.02, 0.28)	–	–
	Overall (raw score)	1.13	(0.08, 2.19)	1.04	(0.06, 2.01)	0.73	(-0.15, 1.60)	0.75	(-0.13, 1.63)	–	–
Current intensity of indoor solid fuel use	Visuospatial/ Executive	-0.05	(-0.10, 0.00)	-0.03	(-0.07, 0.01)	-0.01	(-0.05, 0.03)	-0.01	(-0.05, 0.04)	–	–
	Naming	-0.01	(-0.06, 0.04)	0.00	(-0.05, 0.04)	0.00	(-0.04, 0.05)	-0.01	(-0.05, 0.04)	–	–
	Attention	-0.07	(-0.12, -0.03)	-0.06	(-0.10, -0.02)	-0.05	(-0.09, -0.01)	-0.05	(-0.09, -0.01)	–	–
	Language	-0.03	(-0.07, 0.02)	-0.02	(-0.06, 0.03)	-0.01	(-0.05, 0.03)	-0.01	(-0.04, 0.03)	–	–
	Abstraction	-0.05	(-0.11, 0.00)	-0.05	(-0.10, 0.00)	-0.04	(-0.09, 0.01)	-0.05	(-0.10, 0.00)	–	–
	Delayed recall	-0.03	(-0.08, 0.02)	-0.01	(-0.06, 0.03)	0.00	(-0.05, 0.05)	-0.01	(-0.06, 0.04)	–	–
	Orientation	-0.01	(-0.05, 0.04)	0.00	(-0.05, 0.04)	0.01	(-0.04, 0.05)	-0.01	(-0.05, 0.03)	–	–
	Overall	-0.05	(-0.10, -0.01)	-0.04	(-0.08, 0.01)	-0.02	(-0.06, 0.02)	-0.03	(-0.07, 0.01)	–	–
	Overall (raw score)	-0.31	(-0.58, -0.03)	-0.21	(-0.46, 0.04)	-0.12	(-0.34, 0.11)	-0.19	(-0.41, 0.04)	–	–
Long-term intensity of indoor solid fuel use	Visuospatial/ Executive	-0.08	(-0.14, -0.02)	-0.05	(-0.11, 0.00)	-0.03	(-0.09, 0.02)	-0.02	(-0.08, 0.04)	–	–
	Naming	-0.04	(-0.10, 0.02)	-0.02	(-0.08, 0.04)	-0.02	(-0.08, 0.04)	-0.04	(-0.10, 0.02)	–	–
	Attention	-0.09	(-0.15, -0.03)	-0.06	(-0.12, 0.00)	-0.06	(-0.11, -0.01)	-0.07	(-0.12, -0.01)	–	–
	Language	-0.06	(-0.12, -0.01)	-0.04	(-0.10, 0.01)	-0.04	(-0.09, 0.01)	-0.04	(-0.09, 0.01)	–	–
	Abstraction	-0.05	(-0.12, 0.01)	-0.03	(-0.10, 0.03)	-0.03	(-0.09, 0.04)	-0.04	(-0.11, 0.02)	–	–
	Delayed recall	-0.04	(-0.10, 0.03)	0.00	(-0.07, 0.06)	0.01	(-0.05, 0.07)	0.00	(-0.06, 0.07)	–	–
	Orientation	-0.03	(-0.09, 0.03)	-0.02	(-0.07, 0.04)	-0.01	(-0.07, 0.04)	-0.02	(-0.08, 0.03)	–	–
	Overall	-0.09	(-0.14, -0.03)	-0.05	(-0.10, 0.01)	-0.04	(-0.09, 0.01)	-0.05	(-0.10, 0.00)	–	–
	Overall (raw score)	-0.50	(-0.84, -0.15)	-0.29	(-0.61, 0.03)	-0.22	(-0.51, 0.07)	-0.29	(-0.58, 0.01)	–	–

[a] Difference in score in each cognitive measure associated with a 100-µg/m^3 increase in exposure to $PM_{2.5}$ (n=355), 1-µg/m^3 increase in exposure to black carbon (n=355), use of clean fuel exclusively for cooking or heating versus use of solid fuel as reference (n=400), per 100-day increase in solid fuel stove-use days in the past year (current use; n=400), or per 5-year increase in solid fuel stove-use years over the past 20 years (long-term use; n=394).
Abbreviations: $PM_{2.5}$, particulate matter ≤2.5 µm in aerodynamic diameter; CI, confidence interval.

Supplementary Table 3. Assessment of potential effect modification by province and gender on the associations between measures of cognitive function and personal exposures to air pollution, current indoor fuel use patterns, or intensity of indoor solid fuel use: results from multivariable models.

Effect modifier evaluated	By province			By gender		
	Beijing	Shanxi	Interaction p-value[a]	Male	Female	Interaction p-value[a]
Difference in score and 95% CI per 100-μg/m^3 increase in $PM_{2.5}$ (n = 355)						
Visuospatial/Executive	-0.27 (-0.54, 0.00)	-0.14 (-0.39, 0.11)	0.48	-0.14 (-0.37, 0.08)	-0.31 (-0.64, 0.01)	0.38
Naming	-0.11 (-0.40, 0.17)	-0.16 (-0.42, 0.10)	0.83	-0.08 (-0.32, 0.15)	-0.26 (-0.60, 0.08)	0.38
Attention	-0.47 (-0.73, -0.20)	-0.05 (-0.28, 0.19)	0.02	-0.13 (-0.35, 0.09)	-0.46 (-0.77, -0.15)	0.08
Language	-0.31 (-0.56, -0.05)	-0.06 (-0.28, 0.17)	0.13	-0.15 (-0.36, 0.06)	-0.20 (-0.50, 0.10)	0.81
Abstraction	-0.24 (-0.55, 0.07)	-0.09 (-0.37, 0.18)	0.47	-0.31 (-0.56, -0.06)	0.20 (-0.16, 0.56)	0.02
Delayed recall	-0.28 (-0.58, 0.02)	-0.07 (-0.33, 0.20)	0.28	-0.18 (-0.42, 0.07)	-0.13 (-0.48, 0.22)	0.83
Orientation	-0.25 (-0.51, 0.01)	0.00 (-0.24, 0.23)	0.15	-0.16 (-0.37, 0.06)	-0.01 (-0.32, 0.29)	0.44
Overall	-0.42 (-0.66, -0.18)	-0.10 (-0.31, 0.12)	0.04	-0.22 (-0.42, -0.03)	-0.28 (-0.56, 0.00)	0.74
Overall (raw score)	-2.45 (-3.85, -1.06)	-0.58 (-1.83, 0.67)	0.04	-1.31 (-2.45, -0.16)	-1.64 (-3.29, 0.01)	0.74
Difference in score and 95% CI per 1-μg/m^3 increase in black carbon (n = 355)						
Visuospatial/Executive	-0.08 (-0.21, 0.04)	-0.17 (-0.37, 0.03)	0.47	-0.08 (-0.22, 0.06)	-0.14 (-0.30, 0.03)	0.62
Naming	-0.02 (-0.16, 0.11)	-0.03 (-0.25, 0.19)	0.95	0.02 (-0.13, 0.17)	-0.09 (-0.26, 0.09)	0.36
Attention	-0.18 (-0.30, -0.05)	-0.02 (-0.22, 0.18)	0.17	-0.09 (-0.23, 0.05)	-0.20 (-0.36, -0.04)	0.30
Language	-0.09 (-0.21, 0.03)	0.01 (-0.17, 0.18)	0.37	-0.06 (-0.19, 0.07)	-0.05 (-0.20, 0.10)	0.92
Abstraction	0.00 (-0.14, 0.14)	-0.08 (-0.30, 0.14)	0.56	-0.09 (-0.25, 0.07)	0.08 (-0.10, 0.26)	0.16
Delayed recall	-0.08 (-0.22, 0.05)	0.09 (-0.13, 0.32)	0.18	-0.08 (-0.23, 0.07)	0.02 (-0.16, 0.20)	0.38
Orientation	-0.11 (-0.23, 0.01)	-0.01 (-0.19, 0.18)	0.36	-0.13 (-0.26, 0.00)	-0.01 (-0.16, 0.15)	0.22
Overall	-0.14 (-0.25, -0.02)	-0.04 (-0.22, 0.14)	0.36	-0.11 (-0.23, 0.02)	-0.11 (-0.26, 0.03)	0.96
Overall (raw score)	-0.79 (-1.44, -0.14)	-0.23 (-1.28, 0.82)	0.36	-0.63 (-1.35, 0.09)	-0.66 (-1.50, 0.19)	0.96
Difference in score and 95% CI for exclusive use of clean fuel (ref. use of solid fuel indoors) for cooking (n = 400)						
Visuospatial/Executive	-0.05 (-0.31, 0.21)	-0.04 (-0.28, 0.20)	0.98	-0.08 (-0.34, 0.18)	-0.03 (-0.26, 0.21)	0.77
Naming	-0.16 (-0.41, 0.10)	0.19 (-0.09, 0.47)	0.07	0.00 (-0.28, 0.27)	0.00 (-0.24, 0.25)	0.96
Attention	0.14 (-0.09, 0.38)	0.24 (-0.01, 0.50)	0.57	0.14 (-0.11, 0.40)	0.23 (0.00, 0.45)	0.61
Language	0.01 (-0.22, 0.25)	0.03 (-0.17, 0.24)	0.90	-0.13 (-0.36, 0.10)	0.15 (-0.06, 0.36)	0.08
Abstraction	0.03 (-0.25, 0.30)	0.41 (0.11, 0.70)	0.07	0.34 (0.04, 0.64)	0.04 (-0.22, 0.31)	0.12
Delayed recall	-0.14 (-0.41, 0.13)	0.21 (-0.09, 0.51)	0.09	0.05 (-0.25, 0.34)	-0.02 (-0.28, 0.24)	0.73
Orientation	-0.13 (-0.36, 0.11)	0.01 (-0.25, 0.26)	0.44	0.09 (-0.16, 0.34)	-0.21 (-0.43, 0.02)	0.07
Overall	-0.07 (-0.28, 0.15)	0.22 (-0.03, 0.46)	0.09	0.07 (-0.17, 0.30)	0.05 (-0.16, 0.26)	0.90
Overall (raw score)	-0.39 (-1.65, 0.87)	1.26 (-0.15, 2.67)	0.09	0.39 (-0.98, 1.75)	0.28 (-0.94, 1.50)	0.90

Difference in score and 95% CI for exclusive use of clean fuel (ref. use of solid fuel indoors) for heating (n = 400)						
Visuospatial/Executive	0.11 (-0.15, 0.37)	0.13 (-0.11, 0.37)	0.91	0.11 (-0.16, 0.38)	0.13 (-0.10, 0.36)	0.91
Naming	-0.03 (-0.29, 0.23)	0.21 (-0.04, 0.45)	0.19	-0.05 (-0.32, 0.23)	0.19 (-0.04, 0.42)	0.19
Attention	-0.05 (-0.29, 0.19)	0.12 (-0.11, 0.35)	0.31	0.00 (-0.25, 0.25)	0.06 (-0.15, 0.28)	0.70
Language	-0.01 (-0.24, 0.23)	0.00 (-0.21, 0.22)	0.96	-0.11 (-0.35, 0.13)	0.08 (-0.12, 0.29)	0.22
Abstraction	-0.01 (-0.29, 0.27)	0.12 (-0.15, 0.39)	0.52	-0.29 (-0.59, 0.00)	0.30 (0.05, 0.55)	0.00
Delayed recall	-0.05 (-0.33, 0.23)	0.16 (-0.10, 0.43)	0.27	0.09 (-0.20, 0.38)	0.04 (-0.21, 0.29)	0.81
Orientation	0.24 (0.00, 0.47)	0.07 (-0.15, 0.29)	0.32	0.17 (-0.08, 0.42)	0.13 (-0.08, 0.35)	0.84
Overall	0.06 (-0.16, 0.28)	0.19 (-0.02, 0.40)	0.38	0.06 (-0.17, 0.29)	0.18 (-0.02, 0.38)	0.42
Overall (raw score)	0.33 (-0.94, 1.61)	1.13 (-0.09, 2.36)	0.38	0.33 (-1.01, 1.67)	1.05 (-0.09, 2.19)	0.42
Difference in score and 95% CI per 100-day increase in solid fuel stove-use days in the past year (n = 400)						
Visuospatial/Executive	-0.01 (-0.08, 0.05)	0.00 (-0.05, 0.05)	0.72	0.00 (-0.06, 0.06)	-0.01 (-0.07, 0.05)	0.84
Naming	0.02 (-0.04, 0.09)	-0.04 (-0.10, 0.03)	0.17	-0.01 (-0.08, 0.05)	0.00 (-0.06, 0.05)	0.84
Attention	-0.05 (-0.11, 0.01)	-0.05 (-0.11, 0.01)	0.99	-0.03 (-0.09, 0.03)	-0.07 (-0.12, -0.01)	0.32
Language	0.00 (-0.06, 0.06)	-0.01 (-0.05, 0.04)	0.90	0.03 (-0.03, 0.08)	-0.03 (-0.08, 0.02)	0.13
Abstraction	-0.01 (-0.08, 0.06)	-0.09 (-0.16, -0.03)	0.09	-0.07 (-0.15, 0.00)	-0.04 (-0.10, 0.02)	0.43
Delayed recall	0.03 (-0.04, 0.10)	-0.05 (-0.12, 0.01)	0.09	-0.04 (-0.11, 0.03)	0.01 (-0.05, 0.07)	0.28
Orientation	0.00 (-0.06, 0.06)	-0.01 (-0.06, 0.05)	0.87	-0.05 (-0.11, 0.01)	0.03 (-0.02, 0.08)	0.04
Overall	-0.01 (-0.06, 0.05)	-0.06 (-0.11, 0.00)	0.20	-0.04 (-0.10, 0.02)	-0.03 (-0.07, 0.02)	0.70
Overall (raw score)	-0.04 (-0.36, 0.28)	-0.32 (-0.63, -0.01)	0.20	-0.23 (-0.56, 0.10)	-0.15 (-0.43, 0.12)	0.70
Difference in score and 95% CI per 5-year increase in solid fuel stove-use years over the past 20 years (n = 394)						
Visuospatial/Executive	-0.05 (-0.12, 0.02)	0.03 (-0.06, 0.12)	0.18	-0.02 (-0.11, 0.06)	-0.02 (-0.10, 0.05)	0.96
Naming	-0.05 (-0.12, 0.02)	-0.02 (-0.13, 0.08)	0.66	-0.07 (-0.16, 0.02)	-0.02 (-0.10, 0.05)	0.47
Attention	-0.09 (-0.15, -0.02)	-0.02 (-0.12, 0.08)	0.28	-0.05 (-0.14, 0.03)	-0.08 (-0.15, -0.01)	0.65
Language	-0.06 (-0.12, 0.00)	-0.01 (-0.09, 0.07)	0.32	-0.03 (-0.11, 0.05)	-0.05 (-0.12, 0.02)	0.65
Abstraction	-0.03 (-0.11, 0.05)	-0.07 (-0.19, 0.05)	0.56	-0.08 (-0.18, 0.02)	-0.02 (-0.10, 0.06)	0.39
Delayed recall	0.00 (-0.07, 0.08)	0.00 (-0.11, 0.12)	0.99	-0.05 (-0.14, 0.05)	0.03 (-0.05, 0.11)	0.19
Orientation	-0.03 (-0.09, 0.04)	-0.02 (-0.12, 0.07)	0.97	-0.05 (-0.13, 0.03)	-0.01 (-0.08, 0.06)	0.40
Overall	-0.06 (-0.12, 0.00)	-0.03 (-0.12, 0.06)	0.67	-0.08 (-0.15, 0.00)	-0.03 (-0.10, 0.03)	0.36
Overall (raw score)	-0.33 (-0.68, 0.02)	-0.19 (-0.72, 0.34)	0.67	-0.44 (-0.89, 0.01)	-0.19 (-0.56, 0.18)	0.36

[a] Interaction p-values were derived by including each potential modifier as an interaction term with exposure variables in our models.
Abbreviations: CI, confidence interval.

Supplementary Table 4. Assessment of potential effect modification by education level on the associations between measures of cognitive function and personal exposures to air pollution, current indoor fuel use patterns, or intensity of indoor solid fuel use: results from multivariable models.

	Difference in score and 95% CI			Interaction p-value[a]		
	No school	Primary school	Secondary school/ college	Exposure:Primary school (ref. no school)	Exposure:Secondary school/college (ref. no school)	Exposure:Secondary school/college (ref. primary school)
$PM_{2.5}$ (per 100-μg/m^3; n=355)						
Visuospatial/Executive	-0.28 (-0.92, 0.36)	-0.09 (-0.37, 0.20)	-0.27 (-0.51, -0.02)	1.00	1.00	0.97
Naming	-0.47 (-1.15, 0.21)	-0.11 (-0.41, 0.19)	-0.12 (-0.38, 0.14)	1.00	0.98	1.00
Attention	-0.87 (-1.49, -0.25)	-0.24 (-0.51, 0.04)	-0.16 (-0.40, 0.08)	0.19	0.10	1.00
Language	0.14 (-0.46, 0.73)	-0.38 (-0.65, -0.12)	-0.04 (-0.27, 0.18)	0.33	1.00	0.14
Abstraction	-0.44 (-1.16, 0.29)	-0.11 (-0.43, 0.21)	-0.16 (-0.44, 0.11)	1.00	1.00	1.00
Delayed recall	0.20 (-0.50, 0.90)	-0.36 (-0.66, -0.05)	-0.06 (-0.33, 0.20)	0.44	1.00	0.41
Orientation	-0.24 (-0.85, 0.37)	-0.02 (-0.30, 0.25)	-0.16 (-0.39, 0.07)	1.00	1.00	1.00
Overall	-0.34 (-0.91, 0.23)	-0.28 (-0.53, -0.03)	-0.20 (-0.42, 0.01)	1.00	1.00	1.00
Overall (raw score)	-1.99 (-5.29, 1.31)	-1.63 (-3.09, -0.17)	-1.18 (-2.44, 0.08)	1.00	1.00	1.00
Black carbon (per 1-μg/m^3; n=355)						
Visuospatial/Executive	-0.12 (-0.40, 0.17)	-0.09 (-0.26, 0.09)	-0.11 (-0.26, 0.03)	0.98	1.00	0.97
Naming	-0.15 (-0.45, 0.16)	0.03 (-0.15, 0.22)	-0.04 (-0.19, 0.12)	0.56	0.79	0.83
Attention	-0.42 (-0.70, -0.15)	-0.11 (-0.28, 0.06)	-0.08 (-0.22, 0.05)	0.13	0.07	0.96
Language	-0.13 (-0.39, 0.14)	-0.10 (-0.26, 0.07)	-0.02 (-0.16, 0.11)	0.98	0.76	0.77
Abstraction	-0.17 (-0.49, 0.15)	0.14 (-0.05, 0.34)	-0.10 (-0.26, 0.06)	0.21	0.92	0.13
Delayed recall	-0.12 (-0.43, 0.19)	0.00 (-0.19, 0.19)	-0.04 (-0.20, 0.11)	0.79	0.90	0.94
Orientation	-0.20 (-0.46, 0.07)	-0.14 (-0.30, 0.03)	-0.02 (-0.16, 0.12)	0.92	0.47	0.52
Overall	-0.30 (-0.55, -0.05)	-0.08 (-0.24, 0.07)	-0.09 (-0.21, 0.04)	0.30	0.29	1.00
Overall (raw score)	-1.74 (-3.20, -0.27)	-0.47 (-1.37, 0.44)	-0.51 (-1.25, 0.23)	0.30	0.29	1.00
Exclusive use of clean fuel (ref. use of solid fuel indoors) for cooking (n=400)						
Visuospatial/Executive	0.02 (-0.41, 0.44)	0.02 (-0.24, 0.29)	-0.17 (-0.44, 0.10)	1.00	1.00	0.96
Naming	0.14 (-0.29, 0.57)	0.02 (-0.26, 0.30)	-0.07 (-0.34, 0.21)	1.00	1.00	1.00
Attention	0.14 (-0.26, 0.54)	0.29 (0.03, 0.55)	0.12 (-0.14, 0.38)	1.00	1.00	1.00
Language	-0.10 (-0.48, 0.29)	0.18 (-0.06, 0.41)	-0.09 (-0.33, 0.15)	0.69	1.00	0.37
Abstraction	0.08 (-0.39, 0.54)	0.31 (0.00, 0.62)	0.09 (-0.21, 0.40)	1.00	1.00	0.89
Delayed recall	0.14 (-0.32, 0.59)	0.07 (-0.23, 0.38)	-0.09 (-0.39, 0.21)	1.00	1.00	1.00
Orientation	-0.55 (-0.94, -0.16)	0.13 (-0.13, 0.38)	-0.07 (-0.33, 0.18)	0.01	0.13	0.77
Overall	-0.03 (-0.40, 0.33)	0.18 (-0.06, 0.42)	-0.02 (-0.26, 0.22)	0.95	1.00	0.69
Overall (raw score)	-0.20 (-2.33, 1.92)	1.05 (-0.36, 2.46)	-0.11 (-1.49, 1.27)	0.95	1.00	0.69

Exclusive use of clean fuel (ref. use of solid fuel indoors) for heating (n=400)						
Visuospatial/Executive	0.16 (-0.32, 0.63)	0.16 (-0.11, 0.44)	0.07 (-0.20, 0.33)	1.00	1.00	1.00
Naming	0.12 (-0.36, 0.59)	0.05 (-0.22, 0.33)	0.12 (-0.14, 0.39)	1.00	1.00	1.00
Attention	-0.05 (-0.49, 0.39)	0.06 (-0.20, 0.32)	0.05 (-0.20, 0.29)	1.00	1.00	1.00
Language	0.03 (-0.40, 0.46)	0.01 (-0.23, 0.26)	-0.03 (-0.26, 0.21)	1.00	1.00	1.00
Abstraction	-0.27 (-0.78, 0.24)	0.12 (-0.18, 0.42)	0.11 (-0.18, 0.40)	0.59	0.61	1.00
Delayed recall	-0.13 (-0.63, 0.37)	0.11 (-0.18, 0.41)	0.07 (-0.21, 0.35)	1.00	1.00	1.00
Orientation	0.39 (-0.04, 0.82)	0.22 (-0.03, 0.47)	0.01 (-0.23, 0.24)	1.00	0.37	0.64
Overall	0.03 (-0.36, 0.43)	0.19 (-0.05, 0.42)	0.11 (-0.11, 0.33)	1.00	1.00	1.00
Overall (raw score)	0.19 (-2.12, 2.49)	1.08 (-0.28, 2.44)	0.63 (-0.66, 1.93)	1.00	1.00	1.00
Current intensity of indoor solid fuel use (per 100-day increase in solid fuel stove-use days in the past year; n=400)						
Visuospatial/Executive	-0.04 (-0.15, 0.07)	-0.02 (-0.08, 0.04)	0.03 (-0.04, 0.10)	1.00	0.95	0.90
Naming	-0.03 (-0.14, 0.09)	-0.01 (-0.07, 0.05)	0.00 (-0.06, 0.07)	1.00	1.00	1.00
Attention	-0.07 (-0.18, 0.03)	-0.06 (-0.12, 0.00)	-0.03 (-0.09, 0.04)	1.00	1.00	1.00
Language	0.04 (-0.06, 0.14)	-0.03 (-0.08, 0.03)	0.00 (-0.05, 0.06)	0.71	1.00	1.00
Abstraction	-0.01 (-0.13, 0.12)	-0.07 (-0.13, 0.00)	-0.05 (-0.13, 0.02)	1.00	1.00	1.00
Delayed recall	-0.02 (-0.14, 0.10)	-0.02 (-0.08, 0.05)	0.00 (-0.08, 0.07)	1.00	1.00	1.00
Orientation	0.09 (-0.02, 0.19)	-0.04 (-0.10, 0.01)	0.01 (-0.05, 0.08)	0.07	0.67	0.54
Overall	-0.01 (-0.11, 0.09)	-0.05 (-0.10, 0.01)	-0.02 (-0.08, 0.04)	1.00	1.00	1.00
Overall (raw score)	-0.06 (-0.61, 0.50)	-0.27 (-0.57, 0.03)	-0.12 (-0.47, 0.23)	1.00	1.00	1.00
Long-term intensity of indoor solid fuel use (per 5-year increase in solid fuel stove-use years over the past 20 years; n=394)						
Visuospatial/Executive	-0.11 (-0.24, 0.03)	-0.01 (-0.10, 0.08)	0.02 (-0.07, 0.10)	0.80	0.40	1.00
Naming	-0.09 (-0.23, 0.05)	-0.03 (-0.12, 0.07)	-0.04 (-0.13, 0.05)	1.00	1.00	1.00
Attention	-0.20 (-0.33, -0.08)	-0.04 (-0.13, 0.05)	-0.03 (-0.12, 0.05)	0.09	0.08	1.00
Language	-0.06 (-0.18, 0.06)	-0.02 (-0.10, 0.06)	-0.05 (-0.13, 0.03)	1.00	1.00	1.00
Abstraction	-0.06 (-0.21, 0.08)	0.03 (-0.07, 0.13)	-0.10 (-0.20, 0.00)	0.89	1.00	0.22
Delayed recall	-0.10 (-0.24, 0.05)	0.03 (-0.07, 0.13)	0.02 (-0.07, 0.12)	0.46	0.52	1.00
Orientation	-0.05 (-0.18, 0.08)	-0.04 (-0.12, 0.05)	0.00 (-0.09, 0.08)	1.00	1.00	1.00
Overall	-0.14 (-0.26, -0.03)	-0.01 (-0.09, 0.07)	-0.04 (-0.12, 0.03)	0.18	0.45	1.00
Overall (raw score)	-0.83 (-1.50, -0.17)	-0.07 (-0.53, 0.39)	-0.25 (-0.69, 0.19)	0.18	0.45	1.00

[a] Interaction p-values were derived by including education as an interaction term with exposure variables in our models. The p-values were adjusted using the Bonferroni method to correct for multiple comparisons.
Abbreviations: CI, confidence interval.

Supplementary Table 5. Sensitivity analyses for the associations between measures of cognitive function and personal exposures to air pollution: results from multivariable models.

Sensitivity analysis	n	Difference in score[a] and 95% CI								
		Visuospatial/ Executive	Naming	Attention	Language	Abstraction	Delayed recall	Orientation	Overall	Overall (raw score)
Personal $PM_{2.5}$										
Adjusted for whether had diabetes	355	-0.20 (-0.39, -0.01)	-0.12 (-0.32, 0.08)	-0.23 (-0.41, -0.04)	-0.17 (-0.35, 0.00)	-0.16 (-0.37, 0.05)	-0.16 (-0.36, 0.05)	-0.10 (-0.28, 0.08)	-0.24 (-0.40, -0.07)	-1.38 (-2.35, -0.41)
Adjusted for whether had heart disease	355	-0.20 (-0.38, -0.01)	-0.14 (-0.34, 0.06)	-0.23 (-0.41, -0.05)	-0.16 (-0.34, 0.01)	-0.16 (-0.37, 0.06)	-0.16 (-0.36, 0.04)	-0.11 (-0.29, 0.07)	-0.24 (-0.40, -0.08)	-1.40 (-2.36, -0.44)
Adjusted for whether had high blood pressure	355	-0.20 (-0.39, -0.01)	-0.14 (-0.34, 0.06)	-0.23 (-0.42, -0.05)	-0.17 (-0.34, 0.01)	-0.16 (-0.38, 0.05)	-0.17 (-0.37, 0.04)	-0.11 (-0.29, 0.07)	-0.25 (-0.41, -0.08)	-1.44 (-2.40, -0.47)
Adjusted for annual household income in more resolved categories[b]	355	-0.22 (-0.41, -0.03)	-0.14 (-0.34, 0.06)	-0.24 (-0.42, -0.06)	-0.16 (-0.34, 0.02)	-0.14 (-0.35, 0.08)	-0.15 (-0.36, 0.05)	-0.14 (-0.32, 0.04)	-0.25 (-0.41, -0.08)	-1.43 (-2.40, -0.46)
Adjusted for body mass index	355	-0.20 (-0.39, -0.01)	-0.13 (-0.33, 0.06)	-0.23 (-0.41, -0.05)	-0.16 (-0.34, 0.01)	-0.16 (-0.37, 0.05)	-0.16 (-0.36, 0.04)	-0.11 (-0.29, 0.07)	-0.24 (-0.40, -0.07)	-1.38 (-2.33, -0.43)
Personal black carbon										
Adjusted for whether had diabetes	355	-0.11 (-0.22, 0.00)	-0.02 (-0.14, 0.10)	-0.13 (-0.24, -0.03)	-0.06 (-0.16, 0.04)	-0.02 (-0.14, 0.10)	-0.04 (-0.16, 0.08)	-0.07 (-0.18, 0.03)	-0.11 (-0.20, -0.01)	-0.63 (-1.19, -0.06)
Adjusted for whether had heart disease	355	-0.11 (-0.22, 0.00)	-0.03 (-0.14, 0.09)	-0.14 (-0.24, -0.03)	-0.06 (-0.16, 0.04)	-0.02 (-0.14, 0.10)	-0.04 (-0.16, 0.08)	-0.08 (-0.18, 0.02)	-0.11 (-0.21, -0.02)	-0.65 (-1.21, -0.09)
Adjusted for whether had high blood pressure	355	-0.10 (-0.21, 0.01)	-0.02 (-0.14, 0.09)	-0.14 (-0.24, -0.03)	-0.06 (-0.16, 0.04)	-0.02 (-0.14, 0.10)	-0.04 (-0.16, 0.08)	-0.08 (-0.18, 0.02)	-0.11 (-0.20, -0.01)	-0.63 (-1.19, -0.06)
Adjusted for annual household income in more resolved categories[b]	355	-0.12 (-0.23, -0.01)	-0.04 (-0.15, 0.08)	-0.14 (-0.24, -0.03)	-0.07 (-0.17, 0.03)	-0.02 (-0.14, 0.10)	-0.03 (-0.15, 0.09)	-0.09 (-0.19, 0.02)	-0.11 (-0.21, -0.02)	-0.66 (-1.23, -0.09)
Adjusted for body mass index	355	-0.10 (-0.21, 0.01)	-0.01 (-0.13, 0.10)	-0.13 (-0.24, -0.02)	-0.05 (-0.15, 0.05)	-0.02 (-0.14, 0.10)	-0.03 (-0.15, 0.09)	-0.07 (-0.18, 0.03)	-0.10 (-0.19, 0.00)	-0.58 (-1.13, -0.02)

[a] Difference in score in each cognitive measure associated with a 100-$\mu g/m^3$ increase in exposure to $PM_{2.5}$ or 1-$\mu g/m^3$ increase in exposure to black carbon.
[b] The original questionnaire collected participants' annual household income in the following categories: less than 2,500 yuan, 2500-4999 yuan, 5000-9999 yuan, 10000-19999 yuan, 20000-34999 yuan, above 35,000 yuan. We assigned participants who did not disclose the income (n=44) as a separate category.
Abbreviations: CI, confidence interval; $PM_{2.5}$, particulate matter ≤2.5 μm in aerodynamic diameter.

Supplementary Table 6. Sensitivity analyses for the associations between measures of cognitive function and intensity of indoor solid fuel use: results from multivariable models.

Sensitivity analysis	n	Difference in score[a] and 95% CI								
		Visuospatial/ Executive	Naming	Attention	Language	Abstraction	Delayed recall	Orientation	Overall	Overall (raw score)
Current intensity of indoor solid fuel use										
Adjusted for annual household income in more resolved categories[b]	400	-0.01 (-0.05, 0.03)	0.00 (-0.05, 0.04)	-0.05 (-0.09, -0.01)	0.00 (-0.04, 0.03)	-0.05 (-0.10, 0.00)	-0.01 (-0.06, 0.04)	-0.01 (-0.05, 0.03)	-0.03 (-0.07, 0.01)	-0.17 (-0.40, 0.05)
Adjusted for whether had diabetes	400	-0.01 (-0.05, 0.03)	0.00 (-0.05, 0.04)	-0.05 (-0.09, -0.01)	-0.01 (-0.04, 0.03)	-0.05 (-0.10, -0.01)	-0.01 (-0.06, 0.04)	0.00 (-0.04, 0.04)	-0.03 (-0.07, 0.01)	-0.19 (-0.41, 0.04)
Adjusted for whether had heart disease	400	-0.01 (-0.05, 0.03)	-0.01 (-0.05, 0.04)	-0.05 (-0.09, -0.01)	-0.01 (-0.04, 0.03)	-0.05 (-0.10, 0.00)	-0.01 (-0.06, 0.04)	-0.01 (-0.05, 0.03)	-0.03 (-0.07, 0.01)	-0.19 (-0.41, 0.03)
Adjusted for whether had high blood pressure	400	-0.01 (-0.05, 0.04)	-0.01 (-0.05, 0.04)	-0.05 (-0.09, -0.01)	-0.01 (-0.04, 0.03)	-0.05 (-0.10, 0.00)	-0.01 (-0.06, 0.04)	-0.01 (-0.05, 0.03)	-0.03 (-0.07, 0.01)	-0.18 (-0.41, 0.04)
Adjusted for body mass index[c]	400	-0.01 (-0.05, 0.04)	0.00 (-0.04, 0.04)	-0.05 (-0.09, -0.01)	0.00 (-0.04, 0.03)	-0.05 (-0.10, 0.00)	-0.01 (-0.06, 0.04)	0.00 (-0.05, 0.04)	-0.03 (-0.07, 0.01)	-0.17 (-0.39, 0.05)
Long-term intensity of indoor solid fuel use										
Adjusted for annual household income in more resolved categories[b]	394	-0.03 (-0.09, 0.03)	-0.04 (-0.10, 0.02)	-0.07 (-0.12, -0.01)	-0.05 (-0.10, 0.01)	-0.05 (-0.12, 0.02)	0.01 (-0.06, 0.07)	-0.02 (-0.08, 0.03)	-0.05 (-0.10, 0.00)	-0.29 (-0.59, 0.01)
Adjusted for whether had diabetes	394	-0.02 (-0.08, 0.04)	-0.04 (-0.10, 0.02)	-0.07 (-0.12, -0.01)	-0.04 (-0.09, 0.01)	-0.04 (-0.11, 0.02)	0.00 (-0.06, 0.07)	-0.03 (-0.08, 0.03)	-0.05 (-0.10, 0.00)	-0.29 (-0.59, 0.01)
Adjusted for whether had heart disease	394	-0.02 (-0.08, 0.04)	-0.04 (-0.10, 0.02)	-0.07 (-0.12, -0.01)	-0.04 (-0.09, 0.01)	-0.04 (-0.11, 0.02)	0.00 (-0.06, 0.06)	-0.03 (-0.08, 0.03)	-0.05 (-0.10, 0.00)	-0.30 (-0.59, 0.00)
Adjusted for whether had high blood pressure	394	-0.02 (-0.08, 0.04)	-0.04 (-0.10, 0.02)	-0.07 (-0.12, -0.01)	-0.04 (-0.09, 0.01)	-0.04 (-0.11, 0.02)	0.00 (-0.06, 0.07)	-0.02 (-0.08, 0.03)	-0.05 (-0.10, 0.00)	-0.28 (-0.58, 0.01)
Adjusted for body mass index[c]	394	-0.02 (-0.08, 0.04)	-0.04 (-0.10, 0.02)	-0.06 (-0.12, -0.01)	-0.04 (-0.09, 0.01)	-0.04 (-0.11, 0.02)	0.00 (-0.06, 0.07)	-0.02 (-0.08, 0.03)	-0.05 (-0.10, 0.00)	-0.27 (-0.57, 0.02)
Excluding 61 participants who reported no history of solid fuel use in the past 20 years	333	-0.03 (-0.09, 0.03)	-0.04 (-0.11, 0.03)	-0.07 (-0.14, -0.01)	-0.05 (-0.11, 0.01)	-0.04 (-0.11, 0.03)	0.00 (-0.07, 0.07)	-0.02 (-0.09, 0.04)	-0.05 (-0.11, 0.01)	-0.29 (-0.61, 0.03)

[a] Difference in score in each cognitive measure associated with a 100-day increase in solid fuel stove-use days in the past year or 5-year increase in solid fuel stove-use years over the past 20 years.

[b] The original questionnaire collected participants' annual household income in the following categories: less than 2,500 yuan, 2500-4999 yuan, 5000-9999 yuan, 10000-19999 yuan, 20000-34999 yuan, above 35,000 yuan. We assigned participants who did not disclose the income (n=49) as a separate category.
[c] We imputed values for participants who did not have body mass index measured (n=16 when investigating current intensity of indoor solid fuel use and n=11 when investigating long-term intensity of indoor solid fuel use).
Abbreviations: CI, confidence interval.

Supplementary Table 7. Sensitivity analyses for the associations between measures of cognitive function and exclusive use of clean fuel (ref. use of solid fuel stoves indoors) currently for cooking or heating: results from multivariable models.

Sensitivity analysis	n	Difference in score[a] and 95% CI								
		Visuospatial/ Executive	Naming	Attention	Language	Abstraction	Delayed recall	Orientation	Overall	Overall (raw score)
Cooking										
Adjusted for heating fuel type	400	-0.06 (-0.24, 0.12)	-0.01 (-0.20, 0.18)	0.19 (0.01, 0.37)	0.02 (-0.13, 0.18)	0.17 (-0.04, 0.39)	0.01 (-0.20, 0.22)	-0.08 (-0.25, 0.09)	0.05 (-0.11, 0.21)	0.29 (-0.67, 1.25)
Adjusted for annual household income in more resolved categories[b]	400	-0.02 (-0.21, 0.16)	-0.02 (-0.21, 0.17)	0.19 (0.01, 0.36)	0.02 (-0.14, 0.19)	0.17 (-0.04, 0.38)	-0.01 (-0.22, 0.19)	-0.08 (-0.25, 0.10)	0.05 (-0.12, 0.21)	0.29 (-0.68, 1.25)
Adjusted for whether had diabetes	400	-0.04 (-0.22, 0.14)	-0.01 (-0.20, 0.18)	0.19 (0.01, 0.37)	0.03 (-0.12, 0.19)	0.18 (-0.03, 0.39)	0.01 (-0.20, 0.22)	-0.08 (-0.26, 0.10)	0.06 (-0.11, 0.22)	0.33 (-0.64, 1.29)
Adjusted for whether had heart disease	400	-0.04 (-0.22, 0.14)	0.00 (-0.19, 0.19)	0.20 (0.02, 0.37)	0.02 (-0.13, 0.18)	0.17 (-0.04, 0.38)	0.01 (-0.19, 0.22)	-0.07 (-0.24, 0.11)	0.06 (-0.10, 0.23)	0.36 (-0.60, 1.31)
Adjusted for whether had high blood pressure	400	-0.05 (-0.22, 0.13)	0.00 (-0.19, 0.19)	0.19 (0.02, 0.37)	0.02 (-0.13, 0.18)	0.18 (-0.03, 0.39)	0.01 (-0.20, 0.22)	-0.07 (-0.25, 0.10)	0.06 (-0.11, 0.22)	0.33 (-0.63, 1.29)
Adjusted for body mass index[c]	400	-0.05 (-0.23, 0.13)	-0.01 (-0.20, 0.17)	0.19 (0.01, 0.36)	0.02 (-0.14, 0.17)	0.17 (-0.04, 0.38)	0.00 (-0.20, 0.21)	-0.08 (-0.25, 0.10)	0.05 (-0.11, 0.21)	0.29 (-0.66, 1.24)
Heating										
Adjusted for cooking fuel type	400	0.12 (-0.05, 0.30)	0.09 (-0.09, 0.27)	0.03 (-0.14, 0.19)	0.00 (-0.16, 0.15)	0.06 (-0.14, 0.25)	0.06 (-0.13, 0.25)	0.15 (-0.01, 0.32)	0.13 (-0.02, 0.28)	0.74 (-0.15, 1.62)
Adjusted for annual household income in more resolved categories[b]	400	0.12 (-0.07, 0.30)	0.06 (-0.12, 0.25)	0.03 (-0.14, 0.21)	0.00 (-0.16, 0.17)	0.05 (-0.15, 0.24)	0.04 (-0.16, 0.23)	0.16 (-0.01, 0.32)	0.12 (-0.04, 0.27)	0.68 (-0.22, 1.57)
Adjusted for whether had diabetes	400	0.13 (-0.05, 0.30)	0.08 (-0.09, 0.26)	0.04 (-0.13, 0.20)	0.01 (-0.15, 0.16)	0.06 (-0.14, 0.26)	0.06 (-0.13, 0.25)	0.14 (-0.02, 0.31)	0.13 (-0.02, 0.28)	0.75 (-0.13, 1.64)
Adjusted for whether had heart disease	400	0.13 (-0.05, 0.30)	0.10 (-0.08, 0.28)	0.05 (-0.11, 0.22)	0.01 (-0.15, 0.17)	0.05 (-0.14, 0.25)	0.07 (-0.12, 0.26)	0.15 (-0.01, 0.32)	0.14 (-0.01, 0.29)	0.82 (-0.06, 1.70)
Adjusted for whether had high blood pressure	400	0.12 (-0.06, 0.30)	0.09 (-0.09, 0.27)	0.04 (-0.13, 0.21)	0.00 (-0.16, 0.16)	0.06 (-0.14, 0.25)	0.06 (-0.13, 0.25)	0.15 (-0.02, 0.31)	0.13 (-0.02, 0.28)	0.74 (-0.14, 1.63)
Adjusted for body mass index[c]	400	0.12 (-0.06, 0.30)	0.09 (-0.09, 0.26)	0.04 (-0.13, 0.20)	0.00 (-0.16, 0.15)	0.06 (-0.14, 0.25)	0.06 (-0.13, 0.25)	0.15 (-0.01, 0.31)	0.13 (-0.02, 0.28)	0.74 (-0.13, 1.62)
Both cooking and heating	400	0.04 (-0.17, 0.25)	0.04 (-0.18, 0.25)	0.12 (-0.08, 0.32)	0.04 (-0.15, 0.22)	0.19 (-0.05, 0.43)	0.01 (-0.22, 0.25)	0.13 (-0.06, 0.32)	0.14 (-0.05, 0.32)	0.80 (-0.27, 1.87)

[a] Difference in score in each cognitive measure associated with using clean fuel exclusively for cooking, heating or both with use of solid fuel indoors as reference.

[b] The original questionnaire collected participants' annual household income in the following categories: less than 2,500 yuan, 2500-4999 yuan, 5000-9999 yuan, 10000-19999 yuan, 20000-34999 yuan, above 35,000 yuan. We assigned participants who did not disclose the income (n=49) as a separate category.
[c] We imputed values for participants who did not have body mass index measured (n=16).
Abbreviations: CI, confidence interval.

Supplementary Table 8. Cognitive equivalence of additional years of age associated with measures of exposure to household air pollution calculated using coefficient estimates from final multivariable models with overall raw MoCA score.

**Exposure**	Estimated cognitive equivalences in years of age[a]
Personal $PM_{2.5}$; n=355	
per 100-μg/m^3 increase	8.29
per IQR (53.2 μg/m^3) increase	4.41
from WHO interim annual target-1 (35 μg/m^3) to participants' mean exposure (101.8 μg/m^3)	5.54
from WHO annual guideline (10 μg/m^3) to participants' mean exposure (101.8 μg/m^3)	7.61
Personal black carbon; n=355	
per 1-μg/m^3 increase	3.65
per IQR (0.9 μg/m^3) increase	3.32
Use of solid fuel for cooking (ref. exclusive use of clean fuel); n=400	2.03
Use of solid fuel for heating (ref. exclusive use of clean fuel); n=400	4.52
Current intensity of indoor solid fuel use; n=400	
per 100-day increase in solid fuel stove-use days in the past year	1.18
Long-term intensity of indoor solid fuel use; n=394	
per 5-year increase in solid fuel stove-use years over the past 20 years	1.91

[a] Estimated cognitive equivalences in years of age were calculated by dividing coefficients for household air pollution exposures by the coefficients for age (range: -0.15 to -0.18) from the final multivariable models predicting overall (raw) cognitive score.

Abbreviations: $PM_{2.5}$, particulate matter ≤2.5 μm in aerodynamic diameter; IQR, interquartile range; CI, confidence interval.

Supplementary Table 9. Baseline health conditions of study participants.

Characteristic	Beijing (n=202)	Shanxi (n=199)	All participants (n=401)
Self-reported physician-diagnosed health conditions; n (%)			
Diabetes	21 (10)	34 (17)	55 (14)
Heart disease	15 (7)	15 (8)	30 (7)
Hypertension	109 (54)	105 (53)	214 (53)
Body mass index (kg/m^2); mean (SD)	26.3 (3.6)	26.0 (3.6)	26.1 (3.6)
Missing; n (%)	9 (5)	7 (4)	16 (4)

Abbreviations: SD, standard deviation.

Supplementary Table 10. Characteristics of participants who reported not having had stroke at enrollment in Beijing and Shanxi sites of the ICP Study by whether they were included in the final analytic sample.

Characteristic	Included in the analytic sample (n=401)	Not included (n=99)	All eligible participants (n=500)
Age (years); mean (SD)	62.5 (8.0)	62.0 (9.3)	62.4 (8.3)
Female; n (%)	232 (58)	55 (56)	287 (57)
Ethnicity; n (%)			
Han	399 (100)	99 (100)	498 (100)
Other	2 (0)	0 (0)	2 (0)
Education level; n (%)			
No school	66 (16)	14 (14)	80 (16)
Primary school	162 (40)	33 (33)	195 (39)
Secondary school/college	173 (43)	52 (53)	225 (45)
Occupation; n (%)			
Agriculture	308 (77)	70 (71)	378 (76)
Other jobs outside of the household	27 (7)	9 (9)	36 (7)
Not working outside of the household	66 (16)	20 (20)	86 (17)
Annual household income (RMB); n (%)			
<2,500	40 (10)	18 (18)	58 (12)
2,500-4,999	33 (8)	6 (6)	39 (8)
5,000-9,999	53 (13)	12 (12)	65 (13)
10,000-19,999	77 (19)	11 (11)	88 (18)
20,000-34,999	74 (18)	21 (21)	95 (19)
≥35,000	75 (19)	14 (14)	89 (18)
Missing	49 (12)	17 (17)	66 (13)
Marital status; n (%)			
Married	362 (90)	86 (87)	448 (90)
Single, widowed, or divorced	39 (10)	13 (13)	52 (10)
Number of household occupants; mean (SD)	3.2 (1.7)	3.1 (1.7)	3.2 (1.7)
Self-reported health status; n (%)			
Excellent	47 (12)	11 (11)	58 (12)
Good	135 (34)	31 (31)	166 (33)
Fair	165 (41)	47 (47)	212 (42)
Poor	54 (13)	10 (10)	64 (13)
Smoking status; n (%)			
Current smoker	95 (24)	32 (32)	127 (25)
Former smoker	65 (16)	11 (11)	76 (15)
Never smoker	241 (60)	56 (57)	297 (59)
Ever lived with a smoker for 6 months[a]; n (%)			
Never	63 (16)	13 (13)	76 (15)
Yes, but not now	66 (16)	11 (11)	77 (15)
Yes, at present	112 (28)	32 (32)	144 (29)
Frequency of exercising; n (%)			
None	168 (42)	45 (45)	213 (43)
Sometimes	100 (25)	26 (26)	126 (25)
Daily	133 (33)	28 (28)	161 (32)

Frequency of drinking alcohol; n (%)			
Never or stopped drinking in past year	258 (64)	61 (62)	319 (64)
Sometimes	100 (25)	21 (21)	121 (24)
Daily	43 (11)	17 (17)	60 (12)
Total cholesterol (mmol/L); mean (SD)	4.7 (1.0)	5.1 (0.8)	4.7 (1.0)
Missing; n (%)	23 (6)	74 (75)	97 (19)
Had experienced past food shortage; n (%)	299 (75)	74 (75)	373 (75)

[a] Only never smokers would report whether they lived with a smoker.

Abbreviations: MoCA, Montreal Cognitive Assessment; SD, standard deviation; RMB, Renminbi.

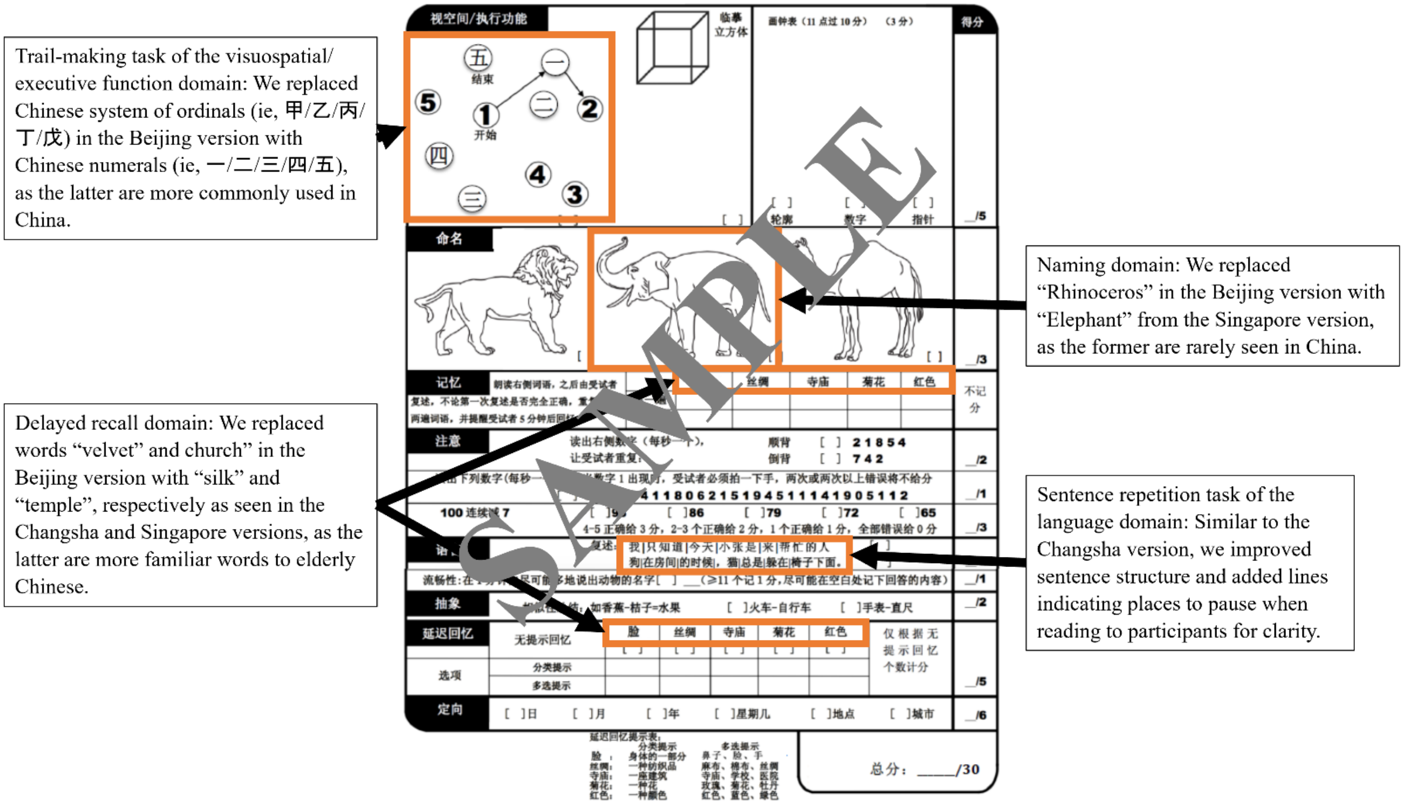

Copyright Z. Nasreddine MD. Reproduced with permission. It is mandatory to follow the online MoCA© Training and Certification Program to administer and score the MoCA©. Copies are available at www.mocatest.org.

Supplementary Fig. 3. Montreal Cognitive Assessment in Mandarin-Chinese used in the ICP Study. Here we illustrate the minor changes made to the Beijing version (https://www.mocatest.org/) of Montreal Cognitive Assessment (MoCA) to increase the cultural appropriateness of the survey for our peri-urban study population.

Supplementary Equation 1. General regression equation for the models used to estimate the cognitive associations with exposures to household air pollution.

$$\gamma_{ij} = \mu + \beta_{ij} + nx_{ij} + \psi_j + \omega_j + \varepsilon_{ij},$$

where γ_{ij} denotes the domain-specific or overall cognitive score for individual i in village j, μ represents a constant; β_{ij} is personal exposure to household air pollution, measured by stove use, $PM_{2.5}$, or black carbon for participant i in village j, x_{ij} represents the set of n covariates, ψ_j is a random intercept at the village level, ω_j represents the random effect, and ε_{ij} is the error term."

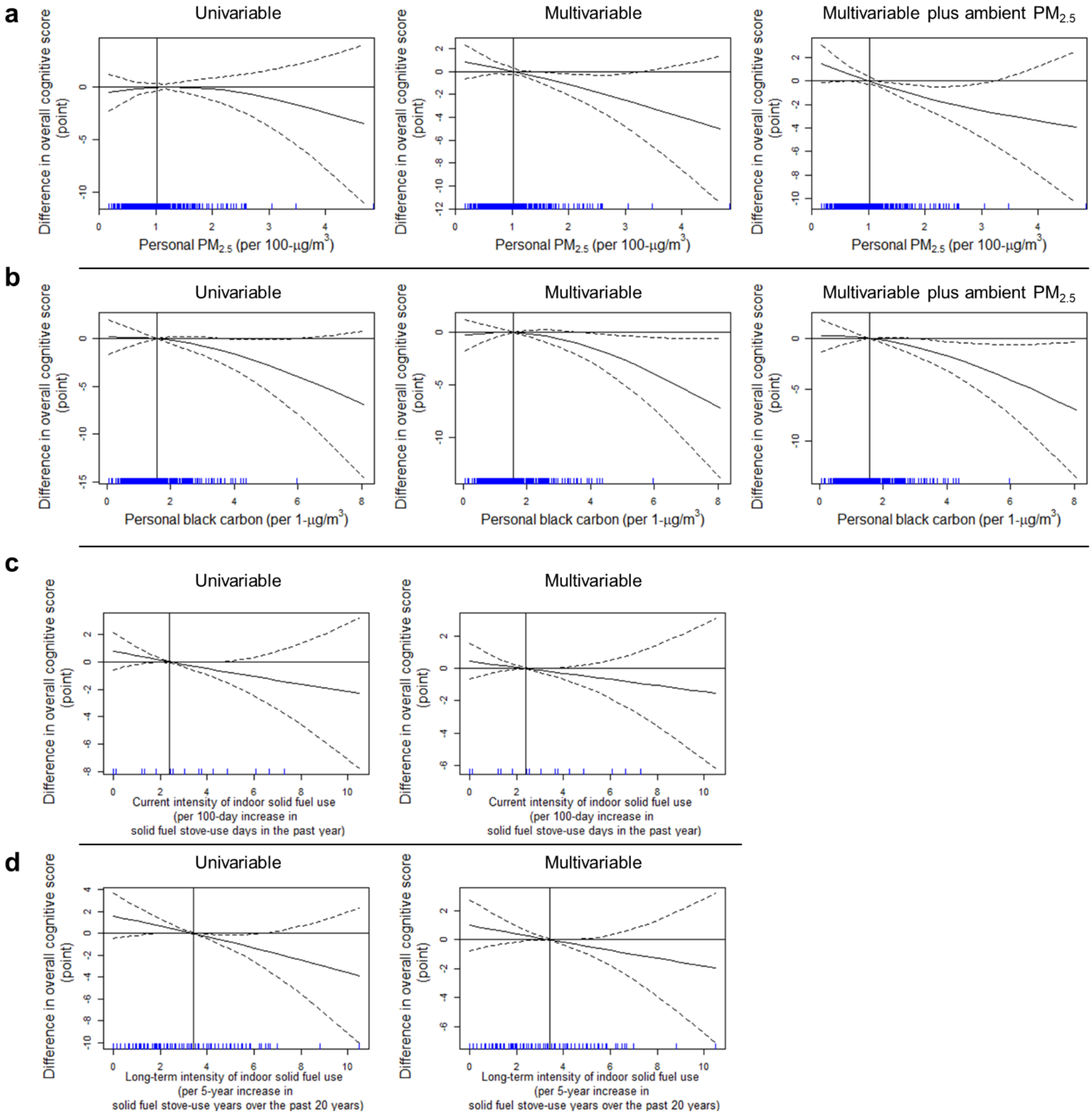

Supplementary Fig. 4. Natural cubic splines with 2 degrees of freedom (solid line) and associated 95% confidence intervals (dashed lines) illustrating the associations between raw score for overall cognition and personal exposures to ***(a)*** $PM_{2.5}$ (n = 355; per 100 µg/m^3) or ***(b)*** black carbon (n = 355; per 1 µg/m^3), ***(c)*** current intensity of indoor solid fuel use (n = 400; per 100-day increase in solid fuel stove-use days in the past year) or ***(d)*** long-term intensity of indoor solid fuel use (n = 394; per 5-year increase in solid fuel stove-use years over the past 20 years) in

older Chinese adults. Mean difference is relative to the mean exposure (vertical black line). Results presented as univariate (left); multivariable models adjusted for province, age, gender, education level, marital status, exposure to tobacco smoking, frequency of farming, frequency of exercising and occupation, annual household income, self-reported health, total cholesterol, past food shortage experience, number of household occupants and frequency of drinking alcohol (middle); and multivariable additionally adjusted for outdoor $PM_{2.5}$ level during exposure assessment (right). The vertical cyan lines along the x-axes show the distribution of the exposures. All models included a random effect at the village level.
Abbreviations: $PM_{2.5}$, particulate matter ≤2.5 µm in aerodynamic diameter.
